# Noise Sources and Strategies for Signal Quality Improvement in Biological Imaging: A Review Focused on Calcium and Cell Membrane Voltage Imaging

**DOI:** 10.3390/bios16010031

**Published:** 2026-01-01

**Authors:** Dmitrii M. Nikolaev, Ekaterina M. Metelkina, Andrey A. Shtyrov, Fanghua Li, Maxim S. Panov, Mikhail N. Ryazantsev

**Affiliations:** 1 Institute of Chemistry, St. Petersburg State University, 7/9 Universitetskaya Emb., Saint Petersburg 199034, Russia; 2 Center for Biophysical Studies, St. Petersburg State Chemical Pharmaceutical University, Professor Popov Str. 14 lit. A, Saint Petersburg 197022, Russia; 3 Nanotechnology Research and Education Centre RAS, Saint Petersburg Academic University, 8/3 Khlopina Street, Saint Petersburg 194021, Russia; 4 National Engineering Research Center for Safe Disposal and Resources Recovery of Sludge, School of Environment, Harbin Institute of Technology, Harbin 150090, China; 5 State Key Laboratory of Urban-rural Water Resource and Environment, School of Environment, Harbin Institute of Technology, Harbin 150090, China; 6 Institute of Biomedical Systems and Biotechnologies, Peter the Great Saint Petersburg Polytechnic University, 29 Polytechnicheskaya Str., Saint Petersburg 195251, Russia

**Keywords:** biological imaging, membrane voltage imaging, optical imaging, cell membrane voltage, signal-to-noise ratio (SNR), genetically encoded voltage indicator (GEVI), calcium-sensitive dye (CSD), genetically encoded calcium indicator (GECI), voltage-sensitive dye (VSD)

## Abstract

This review addresses the challenges of obtaining high-quality quantitative data in the optical imaging of membrane voltage and calcium dynamics. The paper provides a comprehensive overview and systematization of recent studies that analyze factors limiting signal fidelity and propose strategies to enhance data quality. The primary sources of signal degradation in biological optical imaging, with an emphasis on membrane voltage and calcium imaging, are systematically explored across four major indicator classes: voltage-sensitive dyes (VSDs), genetically encoded voltage indicators (GEVIs), calcium-sensitive dyes (CSDs), and genetically encoded calcium indicators (GECIs). Common mechanisms that compromise data quality are classified into three main categories: fundamental photon shot noise, device-related errors, and sample-related measurement errors. For each class of limitation, its physical or biological origin and characteristic manifestations are described, which are followed by an analysis of available mitigation strategies, including hardware optimization, choice of sensors, sample preparation and experimental design, post-processing and computational correction methods.

## 1. Introduction

Biological optical imaging has revolutionized our understanding of living systems and offers valuable insights into cellular and subcellular processes. The visualization of electrical activity in excitable cells and tissues, a task that is especially crucial for neuroscience and cardiology, is currently performed by utilizing calcium and cell membrane voltage imaging techniques [1,2,3,4,5,6]. At the same time, voltage imaging also provides a key to studying inexcitable cells, subcellular events, and slow cellular processes [7]. In calcium and voltage imaging, the reliable extraction of biological information requires the rigorous separation of meaningful signals from experimental noise. This makes the high quality of recorded signals and their careful post-processing crucial for reliable data acquisition. The essential task of improving signal quality and minimizing measurement errors should be considered when choosing a biological sensor and when designing the experimental setup for an imaging experiment. This paper systematically explores the primary factors that degrade signal quality in voltage and calcium imaging and reviews the corresponding mitigation strategies reported to date.

## 2. Fluorescent Indicators for Membrane Voltage and Calcium Imaging

Several methodologies have been proposed to achieve a high-resolution and robust recording of voltage dynamics. Electrophysiological techniques, such as patch-clamp recording and microelectrode arrays, provide direct measurements of membrane potential with high temporal resolution and excellent absolute accuracy, but their invasive nature, limited spatial sampling, and technical complexity restrict their use for subcellular or large-scale in vivo studies [8,9]. In contrast, optical methods offer several advantages, including reduced invasiveness, the ability to analyze numerous cells simultaneously and subcellular compartments, high spatial resolution, and suitability for high throughput in vivo imaging. For optical voltage-related measurements, four major indicator classes are commonly employed:

– **Voltage sensitive dyes (VSDs)** are small, lipophilic organic molecules that become fluorescent after partitioning into the plasma membrane and change their optical properties (typically fluorescence intensity and/or spectrum) in response to changes in membrane potential. VSDs are capable of reporting fast voltage fluctuations, exhibiting response times significantly below 1 ms (often in the tens to hundreds of microseconds range) [10,11]. This rapid response allows them to track neuronal and cardiac action potentials without substantial temporal distortion. At the same time, classical synthetic VSDs have important limitations: they lack intrinsic cell-type specificity and stain essentially all accessible membranes; their high lipophilicity, which is necessary for efficient membrane incorporation, promotes nonspecific binding, can perturb membrane biophysics, and it increases toxicity at the concentrations required for functional imaging. For in vivo applications, an additional practical drawback is the challenge of delivering these hydrophobic dyes selectively and reproducibly to the target region, e.g., a defined brain area [12].
– **Calcium sensitive dyes (CSDs)** are synthetic small molecules that localize to the cytosol or specific organelles and change their fluorescence intensity or spectrum upon binding Ca^2+^. They report the spatiotemporal profile of intracellular Ca^2+^, which is shaped by Ca^2+^ influx through voltage-gated channels, Ca^2+^ release and uptake by internal stores, buffering, and extrusion mechanisms [13,14]. Because membrane depolarization triggers Ca^2+^ influx through voltage-gated calcium channels, calcium transients often follow voltage changes, so CSD fluorescence can serve as an indirect optical readout of electrical activity [15]. However, it should be noted that CSD fluorescence reflects the integrated outcome of multiple Ca^2+^-handling processes rather than voltage dynamics alone. In addition, intracellular calcium transients are generally slower and more prolonged than the underlying membrane voltage changes, so calcium-sensitive dyes behave as low-pass filters that tend to integrate and underestimate rapid spike trains, especially at high firing rates. This temporal integration is particularly evident for somatic Ca^2+^ signals, although appropriately chosen low-affinity, fast indicators can still resolve individual action potentials and many fast synaptic Ca^2+^ transients on the millisecond–tens of milliseconds timescale [16,17,18]. Moreover, small or purely subthreshold depolarizations often produce little or no detectable change in bulk cytosolic Ca^2+^, making calcium dyes insensitive to subthreshold voltage activity.
– **Genetically encoded voltage indicators (GEVIs)** and **genetically encoded calcium indicators (GECIs)** are fluorescent protein-based reporters designed to convert changes in membrane voltage or intracellular Ca^2+^, respectively, into changes in fluorescence. A key advantage of these probes is genetic targetability: by using cell type-specific promoters, they can be selectively expressed in defined cell populations or directed to specific subcellular compartments, enabling optical readouts of voltage or Ca^2+^ dynamics in chosen cells or organelles. Furthermore, both GEVIs and GECIs hold the potential for long-term multicellular recording, providing a means to observe the activity of complex cellular circuits over extended periods [19].

The remainder of this section outlines the key aspects of voltage and calcium indicators. It begins by describing their essential performance criteria, which are crucial for guiding indicator selection. Subsequently, the general measurement modalities (qualitative vs. quantitative) achievable with these probes are detailed, which are followed by a classification of the main indicator types based on their principles of functioning. Examples of commercially available voltage and calcium indicators are given in Table 1 and Table 2.

### 2.1. Performance Metrics for Voltage and Calcium Indicators

Selecting an appropriate optical indicator from the available options demands careful evaluation based on three criteria:

– **Sensitivity** is defined as the magnitude of the indicator’s fluorescence change relative to the change in the measured parameter (e.g., calcium concentration or voltage). A highly sensitive indicator yields a substantial fluorescence response even to minor changes in the target parameter. For voltage indicators, sensitivity is often quantified as the relative change in fluorescence ($\Delta F/F_{0}$) produced by a defined voltage step, commonly 100 mV (for example, from −70 mV to +30 mV). In addition to the magnitude of $\Delta F/F_{0}$, an equally important parameter is the voltage at which the midpoint of the fluorescence–voltage F(V) curve lies. This midpoint determines the voltage range over which the indicator operates in its steep, approximately linear regime. Consequently, optimal probe selection requires matching both the maximal $\Delta F/F_{0}$ and the F(V) midpoint to the expected membrane potential range of the preparation so that the sensor provides a high signal-to-noise ratio over the voltages of interest rather than saturating or operating on the shallow tails of its response curve. For calcium indicators, sensitivity is determined by the change in fluorescence intensity produced by a given fluctuation in Ca^2+^ concentration, for example, which is generated by a single action potential. The calcium dissociation constant ($K_{d}$) sets the concentration range over which an indicator is most responsive [44]. Because the fluorescence–[Ca^2+^] relationship is steepest near $K_{d}$, indicators with low $K_{d}$ values are most sensitive to small changes around resting cytosolic Ca^2+^, whereas higher-$K_{d}$ indicators respond more linearly to large transients without saturation [45]. At the same time, Ca^2+^ affinity strongly influences signal-to-noise and kinetics: low-affinity indicators tend to produce faster signals but with smaller amplitude [18], while high-affinity indicators typically yield larger ΔF/F and better signal-to-noise ratios at the cost of more prolonged, integrative responses [46]. Therefore, the optimal CSD or GECI is chosen by balancing the affinity, dynamic range, and temporal resolution for the specific experimental task.
– **Brightness** quantifies the strength of the fluorescence signal generated by the indicator. It is determined by two main factors: the dye’s efficiency in absorbing photons, quantified by its molar extinction coefficient (ϵ) typically measured at the excitation peak, and its efficiency in emitting photons, which is quantified by the fluorescence quantum yield (FQY)–the ratio of emitted to absorbed photons across the entire emission spectrum. The fluorescence intensity per molecule, serving as the most informative metric for comparing the signal strength of similar indicators, is directly proportional to the product of ϵ and FQY [47]. In voltage and calcium imaging, higher molecular brightness improves the signal-to-noise ratio at a given excitation power, enabling a more reliable detection of small and fast transients. Brighter indicators can also be used with lower illumination intensity, thereby reducing phototoxicity and photobleaching while maintaining the desired measurement quality.
– **Photostability** is the capacity of a fluorophore to withstand repeated excitation–emission cycles without a significant loss of signal over time. It is a critical parameter because photobleaching – the irreversible destruction of fluorophores under illumination—can severely limit the duration and reliability of imaging experiments. Although comprehensive, objective comparisons of photostability across indicators are still limited, newer generations of fluorophores are often specifically engineered to surpass the photostability of their predecessors. In voltage and calcium imaging, higher photostability allows longer recordings, more repeated trials, and stronger illumination (if needed) without rapid signal decay, thereby improving data quality and enabling a stable monitoring of fast or sparse events over extended time scales.

### 2.2. Qualitative and Quantitative Types of Measurement

– Qualitative voltage and calcium measurements track relative changes in the fluorescence signal, rather than calibrated physical units, to report cellular activity. In these experiments, the readout is typically the change in fluorescence intensity normalized by a baseline or mean signal (ΔF/F), which reveals when and where neurons or other types of cells are active and how patterns of activity propagate, but it does not directly yield values such as millivolts or absolute calcium concentration. Because intensiometric measurements depend on variations in factors such as expression level (for GEVIs and GECIs), dye loading (for VSDs and CSDs), illumination power, and photobleaching, qualitative measurements accept these variables as uncontrolled and focus instead on comparing relative signals across time, stimuli, or conditions within the same preparation [47].
– Quantitative measurements with ratiometric voltage and calcium sensors aim to estimate actual changes in membrane potential or Ca^2+^ concentration rather than just relative signal changes. Ratiometric probes operate by simultaneously measuring two distinct fluorescence signals that exhibit counter-directional changes in response to variations in voltage or Ca^2+^ [47]. The fundamental advantage of this approach is that dividing one signal by the other effectively cancels out common confounding factors, such as variations in the indicator concentration, illumination power, or modest sample movement. Achieving true quantitative results requires an additional step of careful calibration. For voltage indicators, this typically involves simultaneous electrophysiological recordings, often using patch-clamp techniques, to correlate the fluorescence ratio with membrane potential [7]. For calcium indicators, calibration can be achieved by determining the indicator’s dissociation constant ($K_{d}$) or by performing in situ titrations [47]. Once calibrated, the fluorescence ratio can be accurately mapped onto physical units, such as millivolts or Ca^2+^ concentration. This rigorous approach enables more accurate and reproducible quantification, facilitating robust comparisons across different cells, experimental preparations, and studies.

### 2.3. Classification of Voltage and Calcium Indicators

**Figure 1:**
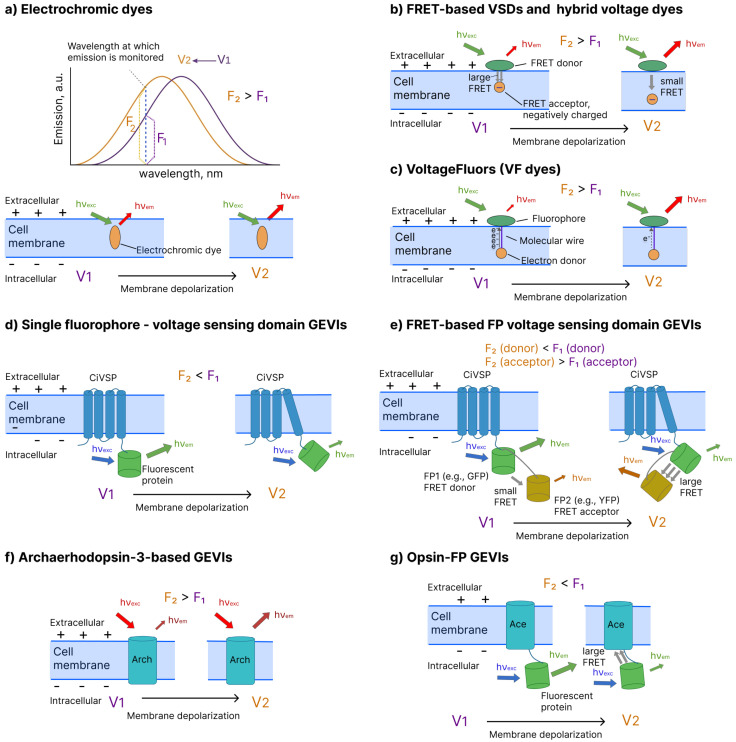
Types of voltage-sensitive dyes (VSDs) and genetically encoded voltage indicators (GEVIs). (**a**) Electrochromic VSDs demonstrate a shift of the absorption/emission band upon membrane polarization/depolarization. (**b**) FRET-based VSDs consist of two components—a fluorescent FRET donor attached at the extracellular side of the plasma membrane and the negatively charged FRET acceptor located inside the membrane. At negative membrane voltage, the acceptor is located close to the donor molecule, and most of the donor emission is transferred to the acceptor via FRET, resulting in a small intensity of donor fluorescence. Upon depolarization, the distance between donor and acceptor increases, reducing FRET and increasing the recorded donor fluorescence. (**c**) VoltageFluors consists of a fluorophore connected to an electron donor through a molecular wire. A fluorophore is attached at the extracellular side of the plasma membrane, and the electron donor is located inside the membrane. At negative voltage, electrons are efficiently transferred from the donor to the fluorophore, quenching its excited state. Membrane depolarization reduces the efficiency of electron transfer, reducing the level of quenching and increasing fluorescence intensity. (**d**) Single fluorophore-voltage-sensing domain GEVIs rely on the voltage-regulated conformation change of the transmembrane voltage-sensitive domain. A fluorescence protein is attached to the domain through a peptide linker; the change in the domain conformation results in the increase/decrease in its fluorescence intensity. (**e**) FRET-based FP voltage-sensing domain GEVIs consist of two fluorescent proteins that act as a FRET donor/acceptor pair attached to a voltage-sensitive domain. Change in the domain conformation results in an increase/decrease in the distance between two FPs and the corresponding decrease/increase in FRET efficiency. (**f**) Archaerhodopsin-3-based sensors are the transmembrane proteins, mutants of archaerhodopsin-3, that possess voltage-dependent fluorescence intensity. (**g**) Opsin-FP sensors consist of a microbial rhodopsin that demonstrates a shift of absorption band upon membrane depolarization, which is attached to a fluorescent protein through a peptide linker. In classical sensors, at negative voltage, the absorption band of the microbial rhodopsin does not overlap with the emission band of the FP, and the intense fluorescence of the FP is recorded. Upon membrane depolarization, the absorption band of the rhodopsin is red-shifted, and it starts overlapping the emission band of the FP. As a result, FP emission is absorbed by microbial rhodopsin through FRET, and decreased FP fluorescence intensity is recorded.

**Figure 2:**
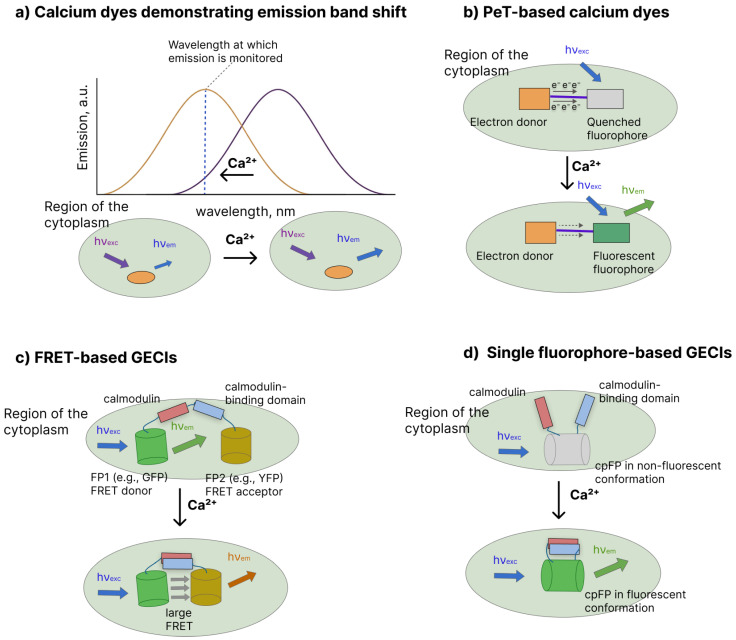
Types of calcium-sensitive dyes (CSDs) and genetically encoded calcium indicators (GECIs). (**a**) Calcium dyes demonstrating emission band shift allow monitoring calcium concentration by recording fluorescence intensity at the specific wavelength, e.g., corresponding to the dye in the calcium-bound conformation. (**b**) Photoinduced electron transfer (PeT)-based calcium dyes consist of the fluorophore moiety and the electron donor moiety. In the ion-free conformation, electron transfer from the donor to the fluorophore efficiently quenches the fluorescence. Upon calcium binding, electron transfer reduces, and an increase in fluorescence intensity is observed. (**c**) FRET-based GECIs consist of the two fluorescent proteins that act as a donor/acceptor FRET pair. The donor FP is attached to the calmodulin, and the acceptor FP is attached to the calmodulin-binding domain. In the ion-free state, the distance between these two FPs is large, and FRET does not occur. Upon calcium binding, the conformational reorganization leads to a decrease in the distance between FPs and increased FRET efficiency. (**d**) Single fluorophore-based GECIs consist of the calmodulin and its binding domain attached to the circularly permuted fluorescent protein (cpFP), and the sensor is not fluorescent in the ion-free conformation. Structural reorganization of the whole complex upon calcium binding leads to the substantial enhancement of cpFP fluorescence intensity.

The following detailed classification of voltage and calcium indicators can be proposed:

– Voltage-sensitive dyes
  – **Electrochromic VSDs**(e.g., di-4-ANNEPS [20], CytoVolt1 [48], ANNIE-6plus [24]) are amphiphilic small molecules that partition into the plasma membrane and sense voltage through electrochromism, i.e., a rapid, linear electric-field-dependent shift in their absorption and/or emission spectra [10,11,49,50]. Both of these effects can be utilized for membrane voltage imaging. For dyes exhibiting a voltage-dependent shift of the fluorescence band, the detection wavelength is chosen on a steep (rising or falling) flank of the emission spectrum, such that even a small voltage-induced spectral displacement redistributes emission power relative to the detection window and yields a detectable increase or decrease in the measured fluorescence intensity (Figure 1a). Dyes exhibiting a voltage-dependent shift of the absorption band operate analogously but at the excitation step rather than emission. In this case, the excitation wavelength is chosen on a steep flank of the absorption spectrum so that spectral displacement alters the spectral overlap between the excitation band and the dye’s absorption band. The voltage-dependent shift therefore changes the fraction of incident photons that are absorbed and promoted to the excited state, producing a corresponding increase or decrease in the measured fluorescence intensity.
    A key advantage of electrochromic VSDs is that they can support ratiometric measurements: two fluorescence signals are recorded at two fixed wavelengths $\lambda_{1}$ and $\lambda_{2}$ positioned on opposite sides of the emission band so that the voltage-induced shift of the spectrum causes counter-directional changes: $F_{1}\left(\lambda_{1}\right)=\kappa_{1}V$ and $F_{2}\left(\lambda_{2}\right)=\kappa_{2}V$. Taking the ratio $R=F_{1}\left(\lambda_{1}\right)/F_{2}\left(\lambda_{2}\right)$ yields a signal that depends on membrane potential ($R=\kappa_{3}V$, where $\kappa_{3}=\kappa_{1}/\kappa_{2}$) but is largely independent of dye concentration, illumination intensity, or photobleaching, because any global scaling factor (for example, dye loss from $c_{A}$ to $c_{B}=\alpha c_{A}$) multiplies both the numerator and denominator and thus cancels out in the ratio. This ratiometric approach reduces artifacts from dye leakage, nonuniform staining, and slow signal drifts, providing a more robust and quantitative readout of membrane voltage than single-channel intensity measurements.
  – **FRET-based VSDs**(e.g., DiO/DPA [51], CC2-DMPE/DiSBAC4(3) [52]) exploit Forster resonance energy transfer, whose efficiency depends steeply on the distance and relative orientation between donor and acceptor fluorophores [53]. In classical implementations, the donor is an immobile, membrane-associated fluorophore, whereas the acceptor is a mobile, negatively charged lipophilic dye (typically an oxonol) that redistributes within the electric field across the membrane. Changes in membrane potential drive the oxonol to move closer to or farther from the donor layer, thereby modulating the donor–acceptor separation and FRET efficiency, which in turn alters both donor quenching and acceptor emission (Figure 1b). As with electrochromic dyes, these probes enable a ratiometric readout by monitoring the ratio of donor and acceptor fluorescence, providing the usual advantages of ratiometry, including reduced sensitivity to variations in dye concentration or illumination intensity.
  – **Hybrid voltage sensors**(hVOS, e.g., hVOS 2.0 [54,55]) use the same FRET principle as small-molecule FRET VSDs but combine a genetically encoded fluorescent protein with a synthetic, voltage-sensing quencher. In most hVOS designs, a membrane-anchored fluorescent protein (e.g., eGFP or other XFPs) acts as the FRET donor, while the negatively charged, lipophilic anion dipicrylamine (DPA) serves as a non-fluorescent acceptor whose membrane distribution depends on the voltage. Changes in membrane potential drive DPA to shuttle between the inner and outer leaflets of the lipid bilayer, thereby changing its distance to the membrane-anchored fluorescent protein and modulating FRET efficiency, which results in voltage-dependent quenching of the donor fluorescence. A key advantage of this chemigenetic architecture is that the spectral properties are largely defined by the choice of fluorescent protein, so hVOS probes can, in principle, be engineered across different spectral ranges, enabling the flexible selection of excitation and emission wavelengths and easier multiplexing with other optical indicators or actuators.
  – **VoltageFluors**(VF dyes, e.g., FluoVolt [25,56], BeRST1 [26]) report changes in membrane potential by modulating the rate of photoinduced electron transfer (PeT) between an electron-rich or electron-poor moiety and a fluorescent reporter embedded in the plasma membrane (Figure 1c). In a typical design, the dye consists of a bright fluorophore linked via a conjugated molecular wire (often phenylenevinylene- or fluorene-based) that spans the membrane to an electron donor (for donor-PeT) or acceptor (for acceptor-PeT). In the resting state, PeT from the donor to the excited fluorophore (or from the excited fluorophore to an acceptor) partially quenches fluorescence. Changes in membrane potential alter the local electric field across the membrane, shifting the PeT efficiency and thereby changing the fluorophore’s quantum yield and fluorescence intensity. VF dyes therefore convert voltage-dependent changes in the PeT efficiency into large, approximately linear changes in fluorescence (often 20–50% ΔF/F per 100 mV) with submillisecond kinetics, enabling an accurate optical recording of action potentials and fast subthreshold voltage dynamics in neurons and other excitable cells [49].
– Genetically Encoded Voltage Indicators
  – **Single-fluorophore voltage-sensing domain GEVIs** (e.g., ArchLight A242 [30], ASAP5 [28], JEDI-1P [57]) constitute a major class of genetically encoded voltage indicators in which a transmembrane voltage sensing domain is directly fused to a fluorescent protein (FP) (Figure 1d). Voltage-dependent conformational rearrangements of the domain are allosterically coupled to changes in FP fluorescence [2]. These constructs most commonly employ the *Ciona intestinalis* voltage-sensing phosphatase (Ci-VSP) as the voltage-sensing module with an FP engineered to report subtle alterations in the chromophore environment. Most members of this family, e.g., ArcLight [30] or ASAP5 [28], exhibit negative-going responses in which membrane depolarization produces a reduction in fluorescence intensity. Some other indicators, such as Marina [58] and FlicR1 [59], display positive-going responses, where depolarization leads to an increase in fluorescence.
  – **FRET-based fluorescent protein (FP) voltage-sensing domain GEVIs** (e.g., Mermaid2 [60], VSFP-Butterfly [61]) typically employ a single voltage-sensing domain fused to a donor–acceptor FP pair (intramolecular FRET design, as shown in Figure 1e) [2]. Voltage-dependent conformational rearrangements of the domain alter the relative distance and/or orientation of the two FPs, modulating the efficiency of FRET and producing counter-directional changes in donor and acceptor fluorescence. More recently, intermolecular FRET-based GEVIs have extended this concept by placing donor and acceptor FPs on separate voltage-sensing domains [62]. This spatial arrangement allows voltage-driven conformational changes of these multiple domains to collectively alter the average donor–acceptor separation and orientation within the membrane. FRET-based GEVIs support ratiometric readout (acceptor/donor or donor/acceptor), which suppresses common noise sources such as excitation intensity fluctuations, nonuniform expression, or sample motion at the cost of increased construct size and optical complexity (two excitation/emission channels are required).
  – **Microbial opsin-based GEVIs** harness the voltage-sensing capabilities of several microbial rhodopsins. These proteins exhibit voltage-dependent changes in their absorption or emission spectra [63]. Specifically, some microbial rhodopsins (e.g., *Acetabularia* rhodopsin [64], *L. maculans* rhodopsin [64]) demonstrate a large shift of the absorption band upon membrane depolarization. In turn, in archaerhodopsin-3 and its mutants, membrane depolarization leads to an increase in fluorescence intensity. These two remarkable effects are exploited in two existing classes of microbial opsin-based GEVIs.
    *Archaerhodopsin-3-based sensors* (e.g., QuasAr6a [65], Archon1 [32]) are derived from archaerhodopsin-3 (Arch), which is a microbial rhodopsin with intrinsically voltage-dependent near-infrared fluorescence that increases with membrane depolarization (Figure 1f). Although wild-type Arch is extremely dim [66], the insertion of amino acid replacements have produced much brighter probes [67]. Prominent examples include representatives from the three families: Archers [68,69,70], QuasArs [31,65], and Archons [32]. These rhodopsin-only GEVIs exhibit large fractional fluorescence changes per 100 mV, submillisecond response kinetics, and high photostability. However, these indicators remain substantially dimmer than FP-based GEVIs [67,71], often requiring stronger excitation and/or more sensitive detectors to achieve an adequate signal-to-noise ratio. In addition, they are also inefficiently excited under two-photon illumination, which limits their utility for deep in vivo imaging compared with many FP-based indicators [72].
    *Opsin–FP eFRET GEVIs* (e.g., VARNAM [73], Ace2N-mNeon [74]) fuse a bright fluorescent protein to a voltage-sensitive microbial rhodopsin whose absorption spectrum shifts with membrane potential (Figure 1g). At negative (resting) membrane voltages in the classical, negative-going designs, the rhodopsin absorbance is significantly blue-shifted from the FP emission, so the FRET is weak and the FP remains bright. Upon membrane depolarization, the rhodopsin absorption band red-shifts and effectively overlaps the FP emission band, enhancing FRET and producing a decrease in FP fluorescence; these indicators therefore report depolarization as a negative-going signal. Recent engineering has inverted this relationship, producing opsin-FP GEVIs (e.g., Positron [75], pAce [34]) with lower fluorescence at rest and positive-going fluorescence changes on depolarization while preserving sensitivity and kinetics. This architecture retains the high brightness advantage of FP-based reporters, as well as the fast kinetics of rhodopsin-based reporters, but it typically sacrifices some of the advantages of the rhodopsin-only sensors, such as high photostability.
  – **Bioluminescent GEVIs** are a unique class of genetically encoded voltage indicators in which light is produced enzymatically by a luciferase–luciferin reaction, eliminating the need for external optical excitation [76,77]. In bioluminescent FRET-type GEVIs, a luciferase (donor) and a fluorescent protein (acceptor) are fused to a single transmembrane voltage-sensing domain. Voltage-dependent conformational changes in the domain modulate the distance and relative orientation of the two proteins, thereby changing bioluminescence resonance energy transfer (BRET) efficiency and donor/acceptor emission intensity. A significant benefit of these sensors is that they do not require external excitation light, thereby eliminating any excitation-dependent background and many phototoxicity concerns. Moreover, because bioluminescent voltage reporters can exhibit very low baseline emission at rest, voltage transients can generate bursts of photons against an almost dark background, offering the potential for superior sensitivity in voltage imaging. However, bioluminescent emission is much dimmer than fluorescence from bright FPs, which typically necessitates highly sensitive cameras and/or longer integration times. An additional practical disadvantage is the requirement to supply an exogenous luciferin substrate to the tissue or organism, complicating delivery and potentially limiting experimental duration as substrate is consumed.

– Calcium-sensitive dyes (CSDs) are small-molecule fluorescent indicators that couple a Ca^2+^-chelating moiety to an organic fluorophore. They are commonly grouped into **ratiometric** indicators, which exhibit Ca^2+^-dependent spectral shifts, and **“single-wavelength” intensiometric** indicators, which show Ca^2+^-dependent changes in fluorescence intensity [15,78].
  – **Ratiometric Ca^2+^ dyes** (e.g., Fura-2 [79] and Indo-1 [79]) display Ca^2+^-induced shifts in excitation (Fura-2) or emission (Indo-1) spectra, enabling ratiometric imaging that corrects for variations in dye concentration or light intensity in a manner analogous to the ratiometric use of electrochromic VSDs (Figure 2a) [15,80]. For example, there is the maximum emission of Indo-1 shifts from 475 nm in the ion-free state to approximately 400 nm when saturated with Ca^2+^, providing a robust ratiometric readout [81].
  – **Non-ratiometric (intensiometric) indicators**(e.g., Fluo-3 [82], Cal-520 [83], Oregon Green BAPTA dyes [46]) rely mainly on a Ca^2+^-induced increase in fluorescence quantum yield mediated by intramolecular photoinduced electron transfer (Figure 2b). In the Ca^2+^-free state, the electron-rich chelator is close to the fluorophore and efficiently quenches its excited state through PeT, keeping the dye weakly fluorescent; Ca^2+^-binding reorganizes the chelator and decreases its electron-donating ability, suppressing PeT and producing a large turn-on in fluorescence intensity.

– Genetically Encoded Calcium Indicators. Many widely used GECIs are built around calmodulin (CaM), which is a soluble Ca^2+^-binding protein found in nearly all cells. In many early and current designs, CaM is paired with a CaM-binding peptide derived from myosin light-chain kinase, although other CaM-binding sequences are also used [84,85]. In the Ca^2+^-free state, CaM and the peptide interact only weakly; Ca^2+^ binding to CaM induces a large conformational change that promotes the tight association between CaM and the peptide, which is then transduced into an optical signal by one or two attached fluorescent proteins. As a reporter, this architecture is implemented either with a FRET donor–acceptor pair (FRET-based GECIs) or with a single fluorescent protein (single-fluorophore GECIs).
  – **FRET-based GECIs** (e.g., cameleons [86] and YC-nano indicators [87]) place a donor fluorescent protein on CaM and an acceptor fluorescent protein on the CaM-binding peptide, forming a FP–CaM–linker–peptide–FP fusion (Figure 2c). Under low Ca^2+^, CaM and the peptide are relatively separated, resulting in low FRET efficiency and a signal dominated by donor emission when the donor is selectively excited. Ca^2+^ binding to CaM triggers CaM–peptide association, shortening the donor–acceptor distance, which increases FRET and is recorded as an increase in acceptor emission relative to donor emission (i.e., a ratiometric change).
  – In **single-fluorophore GECIs** (e.g., GCaMP [41,88,89,90], GECO [91,92], and jGCaMP/ jRGECO [93] families), CaM and a CaM-binding peptide are integrated into the fluorescent protein scaffold (Figure 2d). In most designs, they are fused to the termini of a circularly permuted fluorescent protein (typically cpEGFP or related variants) [94,95] or inserted into an internal loop of the FP [96,97]. In the Ca^2+^-free state, the local structure around the chromophore is configured to favor nonradiative decay and weak fluorescence, whereas Ca^2+^ binding drives CaM–peptide association, reorganizes the chromophore microenvironment, and produces a large increase in fluorescence intensity.

## 3. Signal Quality in Voltage and Calcium Imaging: Determinants and Refinement

In voltage or calcium imaging experiments, the most common objective is to obtain a time-dependent signal that accurately reflects membrane voltage dynamics or changes in calcium concentration in the specific region of interest (e.g., in the neuron’s plasma membrane or cytoplasm). This target signal is initially converted into an optical signal, which is subsequently captured by the detector of a microscope camera and transformed into an electrical signal. The experimental setup and measurement protocols are designed to establish a direct, typically linear, relationship between physical values obtained at each step of experiment: namely, between voltage or calcium concentration and the intensity of emitted light as well as between the number of photons absorbed by a detector and the recorded photocurrent. However, the evaluation of the target biological signal is complicated by various interfering components that arise at each step of the measurement. The quality of image data can be compromised by two main types of deviations:

– **Systematic deviations (systematic offset, bias):** A consistent, predictable offset from the true value that remains stable across measurements taken under the same conditions (e.g., a camera systematic offset or an autofluorescence background signal). This measurement error can be evaluated through preliminary experiments and subsequently calibrated out via subtraction or division. It is convenient to categorize systematic deviations in biological imaging as either device-related (instrumental bias) or sample-related (biological or preparation bias).
– **Stochastic deviations (noise):** Random fluctuations around the true value (e.g., photon shot noise, or dark noise). Noise can be classified into three primary types: fundamental photon shot noise, stemming from the quantum nature of photons; device-related noise, caused by instability in equipment like light sources or camera electronic components; and sample-related noise, which is linked to sample preparation and intrinsic sensor properties. Quantitatively, the noise of each origin can be characterized by the standard deviation (σ) or signal variance ($\sigma^{2}$). Given the independence of all noise sources, the overall signal variance ($\sigma_{\mathrm{total}}^{2}$) equals the sum of the variances produced b y each noise component. The SNR is defined as the ratio of the mean signal magnitude ($\bar{S}$) to the standard deviation of the total noise ($\sigma_{N}$):
  $$\mathrm{SNR}=\frac{\bar{S}}{\sigma_{N}}$$

To refine the target signal, each type of systematic offset and noise can be managed by applying instrumental adjustments, mathematical processing, or a combination of both approaches (Table 3). In addition, the selection or molecular engineering of indicators with improved brightness and sensitivity can substantially enhance the signal-to-noise ratio and reduce the impact of these noise sources.

## 4. Photon Shot Noise and Shot-Noise Limited Regime

Photon shot noise stands out from the rest of the error sources in biological imaging as the fundamental and unavoidable source of noise in any optical measurement, stemming from the quantum nature of light [98]. Due to this quantum effect, the detection of a photon by the camera chip is a stochastic process that follows Poisson statistics. The standard deviation of the Poisson distribution recorded by the chip is proportional to the square root of the number of detected photons ($\sqrt{N}$). Since the measured signal intensity is proportional to N, the resulting SNR due to the shot noise scales N/$\sqrt{N}$ = $\sqrt{N}$. This relation shows the theoretical limit caused by shot noise: the SNR improves only with the square root of the number of collected photons. Consequently, shot noise becomes a critical constraint whenever the number of detected photons is low: for example, in high-speed voltage imaging, two-photon microscopy, or when dim sensors are involved in the measurements (see Table 3) [99,100,101,102]. When dealing with these techniques, special attention is required to ensure operating in the fundamentally constrained shot-noise limited regime but not in the regimes limited by experimental equipment or settings such as read-noise or dark-nose limited regimes.

The **shot-noise limited regime** is defined as the operational state where sufficient photons are collected such that the signal quality (SNR) is governed exclusively by the fundamental, unavoidable randomness of photon detection rather than by correctable instrumental noise sources as readout or dark noise. Operating in this state represents the theoretical and practical optimum for quantitative biological imaging. This regime is also desirable because the photon shot noise precisely follows Poisson statistics ($\mathrm{SNR}=\sqrt{N}$), which allows efficient mathematical modeling and effective data processing. Generally, achieving the shot-noise limit confirms that the experimental setup utilizes the applied light dose as efficiently as possible, thus optimizing the crucial balance between obtaining high-quality data and minimizing damage (phototoxicity and photobleaching) to the biological sample. The conceptions of the shot-noise limited and read-noise limited regimes are detailed further in the “Read Noise” section.

**Hardware-based SNR improvement for photon shot noise.** Due to its fundamental nature, photon shot noise, contrary to other error sources such as the dark noise or readout noise described below, cannot be mitigated by improving technology. However, the microscopy setup significantly affects the SNR for photon shot noise by influencing the total number of photons collected from the sample. *Instrumentation considerations that help to achieve the shot-noise limited regime include the following:*

– **Light sources:** A direct way to increase the photon flux coming to the camera detector is to increase the intensity of the excitation light. However, this solution should be applied accurately to prevent phototoxicity or the acceleration of fluorophore photobleaching.
– **Exposure time:** For a constant photon flux, a longer exposure time leads to a proportionally larger number of accumulated photons per frame, while the signal-to-shot-noise ratio improves in proportion to the square root of the exposure time [103]. However, this solution comes at the cost of decreased temporal resolution, which is often critical for recording fast biological processes.
– **Objective lens:** The objective lens is arguably the most critical optical component determining the efficiency of photon collection and, consequently, the achievable SNR for the photon shot noise limit. The **numerical aperture (NA)**, defined as n·sin(θ) (where *n* is the refractive index of the immersion medium and θ is the half-angle of the maximum cone of light captured by the lens), directly dictates the magnitude of the collected signal. A higher NA lens gathers light from a significantly wider angle, thus capturing a greater number of signal photons within a given exposure time [66,99,104].
**
Optical filters and dichroic mirrors:** Optical filters and dichroic mirrors are essential optical components that significantly impact the SNR for the photon shot noise limit by governing light throughput and background rejection. The use of high-quality excitation and emission filters with high transmission percentages (e.g., >95% transmission) ensures the efficient passage of signal photons through the optical path. Maximizing the transmission of the desired signal while minimizing unwanted background light improves the effective SNR. This prioritization ensures the captured light accurately represents the true biological event, enabling the system to operate closer to the theoretical, shot-noise limited regime where data quality is maximized.
– **Confocal pinhole:** Enlarging the area from which photons are collected, e.g., by increasing the pinhole size of the confocal microscope [105], also yields a larger number of photons per frame but comes at the cost of decreased spatial resolution and an increased interference of background signal collected from out-of-focus regions.
– **Quantum efficiency of the camera:** Quantum efficiency (QE) is defined as the percentage of incident photons that a camera sensor successfully converts into a measurable electrical charge. QE is not a single number but a curve that is wavelength-dependent. Cameras are often optimized for the green/yellow spectrum (500–600 nm), which is common for many popular fluorophores. QE typically drops in the UV and far-red/NIR regions. This value is a critical determinant of the camera’s sensitivity and the achievable SNR. In modern cameras, the QE varies from 80% to >95%. A higher QE is always beneficial for SNR enhancement provided all other factors of the experimental setup remain constant, and the trade-off is primarily a higher price of high-QE cameras, particularly those using back-illuminated sensors.
– **Pixel size:** Larger pixels of a sensor collect more photons during the same exposure time as smaller pixels, assuming a consistent density of incident light. The obvious trade-off, however, is a reduction in spatial resolution.

**Post-processing shot noise mitigation.** Strategies for the separation of photon shot noise from a signal of interest rely on the stochastic nature of this type of noise.

– **Signal averaging (temporal and spatial).** A powerful general strategy is to perform signal averaging, which increases the SNR proportionally to the square root of the number of accumulated measurements. This approach can be implemented in several ways.
  When periodic events are analyzed, temporal averaging can be performed by aggregating data from repeated trials [12,74,106,107]. For non-repeating events, photon counts that originate from a specific pixel or region can be averaged across consecutive frames or time points [108,109,110].
  To perform **spatial averaging**, data from adjacent pixels are combined to mitigate random spatial fluctuations. Modern computational algorithms ensure that such an averaging is performed precisely within the region of interest by utilizing automatic segmentation to accurately define the boundaries and exclude pixels from non-target regions [111,112,113].
– **Frequency-domain approaches.** Another class of denoising strategies is based on the frequency-domain distinction between the low-frequency part of the signal, which is due to biological events, and the high-frequency part of the signal, which is due to shot noise. Random fluctuations with frequencies above a predefined cutoff value can be suppressed using low-pass filters, such as Gaussian or Butterworth filters, which ensure that only the lower-frequency components of the signal pass [71,101]. In principle, the filter cutoff should be set just above the fastest frequency component of the signal that one wishes to preserve, in order to efficiently suppress high-frequency shot noise without distorting the underlying kinetics of the measured biological event. In practice, however, the choice of cutoff frequency should be guided by the experimental goal.
– **Dimensionality reduction methods.** Finally, significant differences in the properties of signal and shot noise enable the application of dimensionality reduction algorithms, such as Principal Component Analysis (PCA) [114,115]. These methods process pixel intensity variations across a series of frames, extracting spatially and temporally coherent changes (the signal) while discarding small, dispersed fluctuations (the shot noise). Several more advanced versions of this method have also been developed, including those that apply deep learning-based approaches [116,117].

**Sensor selection.** The signal-to-shot noise ratio is primarily influenced by two intrinsic parameters of the fluorescent sensor: brightness and sensitivity. Brightness is defined as the total number of photons emitted by a sensor per unit of time. Since the shot noise is proportional to the square root of the number of photons, a sensor with higher brightness has lower noise. Sensitivity is defined as the fractional change in the number of emitted photons in response to a change in the measured physical value (e.g., calcium concentration or membrane voltage). To reliably record the signal of interest, especially those that are rapid or subtle, the change in brightness must be large enough to surpass photon shot noise [102]. This can be achieved by selecting brighter and/or more sensitive sensors. Also, it is essential to consider that the camera’s quantum efficiency, the spectral characteristics of the optical filters and dichroic mirrors, and the spectral characteristics of the employed biological sensor must be carefully matched to optimize system performance and ensure efficient signal detection.

**Photon shot noise in voltage vs. calcium imaging.** Photon shot noise is typically a more prounounced limiting factor for voltage imaging than for calcium imaging, which is largely because of differences in the sensor localization, signal amplitudes and kinetics, and practical brightness.

– In calcium imaging, indicators are usually distributed throughout the cytosolic volume, so a substantial number of fluorophores contribute to the signal from each cell. In contrast, most voltage indicators are confined to the thin plasma membrane, meaning that far fewer molecules occupy the relevant optical volume. As a result, for comparable excitation conditions, substantially fewer photons are emitted and collected from a single cell in voltage imaging than in calcium imaging, which directly increases the relative contribution of photon shot noise.
– Genetically encoded calcium indicators often exhibit large fractional fluorescence changes (ΔF/F) during robust Ca^2+^ transients with some brightening GECIs exceeding 100% ΔF/F [41,90,118]. By comparison, many voltage indicators typically exhibit smaller fractional changes in their fluorescence upon corresponding voltage changes (e.g., action potentials) [28,119,120,121]. Importantly, ΔF/F alone does not determine practical sensitivity: a bright probe with modest ΔF/F can achieve a higher signal-to-noise ratio than a very dim probe with a large ΔF/F, because photon shot noise depends on the absolute photon count. This consideration is particularly relevant for archaerhodopsin-3-based GEVIs, which are substantially dimmer than most GECIs or fluorescent-protein-based GEVIs and therefore require higher excitation intensities or tolerate lower SNRs in order to resolve the same physiological events.
– In many standard calcium-imaging experiments, fluorescence signals are large because the underlying Ca^2+^ transients can represent 5- to 10-fold increases in intracellular concentration in some compartments [122], and brightening GECIs convert these excursions into large ΔF/F responses. In contrast, the membrane potential changes that underlie voltage imaging typically span a narrower range: an action potential involves a change of about 100 mV (from roughly −70 mV to +30 mV), and many subthreshold events are only a few millivolts in amplitude [28]. Consequently, many commonly used voltage indicators are operated over a relatively small dynamic range in membrane potential and often exhibit more modest fractional fluorescence changes under typical conditions.
– A further critical distinction between voltage and calcium imaging is the typical timescales and experimental regimes in which they are applied. Calcium imaging is often used to monitor comparatively slow cellular processes, such as somatic calcium signals associated with neuronal firing, which frequently evolve over hundreds of milliseconds to seconds in population-level recordings. At the same time, localized calcium events (e.g., synaptic or presynaptic transients) can exhibit rise times of only a few milliseconds and therefore may also require high-speed acquisition to be accurately resolved [123]. In contrast, voltage imaging is routinely employed to study much faster electrical phenomena, including individual action potentials and high-frequency spike trains, whose kinetics are inherently sub-millisecond. Reflecting these use cases, many large-scale calcium imaging experiments operate at frame rates on the order of 10–30 Hz [124], whereas direct voltage sensors are commonly used with acquisition rates in the kilohertz range to capture rapid voltage dynamics with sub-millisecond temporal resolution. Given these differences in effective kinetics and typical frame rates, strategies for mitigating photon shot noise necessarily diverge. In calcium imaging, relatively long exposure times can often be employed to accumulate a large number of photons per frame, improving the signal-to-shot-noise ratio without compromising temporal resolution in many applications. By contrast, voltage imaging experiments usually require exposure times of 1 ms or less to preserve the fast temporal structure of the signal, which drastically reduces the number of detected photons per frame and correspondingly amplifies the impact of photon shot noise [125,126].

Taken together—fewer fluorophores in the membrane, smaller ΔF/F in many experiments, lower absolute brightness for some GEVI classes, and the need to preserve fast kinetics—these factors make photon shot noise a more critical limiting factor for the SNR in voltage imaging than in calcium imaging, especially when resolving small subthreshold events or operating at high frame rates.

## 5. Systematic and Stochastic Instrumental Measurement Errors

The first two error sources responsible for the corresponding systematic and stochastic deviation of recorded optical signals are due to the sensor of a microscope camera. In a properly engineered digital camera, these errors are due to the sensor properties rather than the associated system electronic components of the camera. Although both are associated with the camera sensor, they must be addressed separately due to their different origins, occurrence at different stages of image capture, and the necessity for independent management and mitigation protocols. Dark current and dark noise are generated within the sensor material itself and accumulate over the entire exposure time (the time then the shutter is open). The longer the exposure, the more dark current and dark noise are accumulated. Bias offset and read noise originate within the camera’s readout circuitry and occur only once, during the readout phase, after the exposure is finished and the signal is transferred off the pixel. It does not depend on the exposure time.

### 5.1. Dark Current, Dark Noise and Camera Systematic Offset

In semiconductors at finite temperature, charge carriers undergo random thermal motion that produces local microscopic current fluctuations, but the net macroscopic current remains zero in the absence of internal or external electric fields. When the built-in junction fields (and any applied fields) in an image sensor are present, thermally generated carriers are driven and collected even in the dark, giving rise to a net dark current while random fluctuations around this mean current still persist. The dark current increases approximately exponentially with temperature and linearly with exposure (integration) time, and its associated fluctuations, known as dark noise, follow Poisson statistics, so the dark noise scales as the square root of the dark signal, just as for photon shot noise.

Dark current is the primary component of the camera systematic offset. **The camera systematic offset** is defined as a non-zero signal level produced by the camera sensor and associated electronics that is consistent and repeatable across different images taken under identical conditions (temperature, exposure time) in the absence of light. This systematic offset, together with the background signals originating from the sample and the microscope’s optical components, creates a constant baseline value that must be accounted for to accurately measure the fluorescence signal.

**Hardware-based mitigation of dark current and dark noise.** Instrumentation considerations include the following:

– **Cooling camera sensor:** The primary method that can be employed is to cool the camera sensor [127,128,129]. The temperature decrease leads to lowering both the systematic offset (dark current) and dark noise. In typical research-grade neuronal calcium imaging experiments, the use of cooled cameras is standard practice. Neuronal calcium dynamics often involve capturing relatively subtle fluorescence signals over extended periods, making it critical to maximize the signal-to-noise ratio (SNR) for accurate quantitative analysis. Voltage imaging faces even greater SNR challenges due to its faster kinetics (sub-millisecond resolution) and lower-sensitivity indicators. Integrated active cooling in scientific CCD, EM-CCD, and sCMOS cameras is commonly implemented with thermoelectric coolers that use the Peltier effect to extract heat from the sensor [130]. Such Peltier-based systems routinely cool detectors tens of degrees below ambient with typical operating temperatures in the range from 0 °C to −40 °C and −50 °C. In deep-cooled EM-CCD systems, multi-stage Peltier devices operating in a vacuum can reach temperatures of up to −80 °C. Achieving and maintaining these deep-cooled operating points requires the efficient removal of waste heat from the hot side of the TEC—typically via forced-air or liquid cooling and high-quality vacuum insulation. TECs integrated into scientific sCMOS and EM-CCD cameras significantly reduce the dark current and thermal noise. Typical silicon CCD and sCMOS sensors show an exponential decrease in dark current with temperature with the dark current roughly halving for every 5–7 °C of cooling in the range relevant for scientific imaging. For example, measurements on cooled CCDs report dark currents on the order of a few electrons per pixel per second near −20 °C, falling to ≲$0.01\;\;e^{-}{\mathrm{pixel}}^{-1}s^{-1}$ at about −80 °C, implying a reduction by roughly two to three orders of magnitude. The associated dark noise decreases proportional to the square root. Therefore, adjusting the sensor cooling regime ensures stable experimental baselines and enables operation closer to the shot-noise limited regime, preventing even weak biological signals from being masked by camera noise and supporting quantitatively reliable, reproducible measurements over acquisition times of many minutes to hours.
– **Decreasing exposure time:** Decreasing exposure time is another possible solution, but it comes at the cost of increased impact of shot noise [131,132]. Therefore, the exposure time must be carefully optimized to find a balance between the impacts of shot noise and dark noise.
– **Camera sensors gain minimization (if possible):** While gain does not directly prevent dark current generation, some EM-CCD gain processes can amplify the accumulated dark current noise, making careful gain application important.

**Correction of camera systematic offset and dark noise mitigation strategies** The camera systematic offset is removed by employing the master dark frame subtraction method [132,133]. Before the main experiment, a sequence of statistically significant number of individual frames (e.g., 20 to 50) is acquired with the microscope shutter closed but under the same experimental conditions (exposure time, temperature, camera settings) as in the main experiment. The pixel values are averaged across all acquired dark frames to remove the stochastic components of read and dark noise while retaining the fixed systematic offset. This average image is called the master dark frame. Subsequently, this dark frame is subtracted pixel-by-pixel from every raw experimental image to remove the systematic offset. Once this systematic offset has been successfully removed, the remaining signal contains mainly random fluctuations. This residual dark noise, if necessary, can then be further processed using the above-described algorithms.

It is worth noting already at this stage that while the dark current is typically the largest and most temperature-sensitive component of the camera systematic offset, it is not the only one. A below-described fixed electronic bias offset (a constant DC voltage applied to the signal to ensure that all recorded pixel values remain positive and prevent data clipping) is also present in all camera systems. Both are accounted for simultaneously when performing a standard dark frame subtraction using a properly acquired master dark frame.

### 5.2. Bias Offset and Read Noise

Bias offset and read noise are systematic and random deviations of a signal that arises in digital camera sensors at the stage when the electrical charge of a pixel is converted to a digital value [98,131]. Before proceeding to a description of the strategies used for their treatment, the two main types of camera sensors applied in biological imaging are described.

The Charge-Coupled Device (CCD) is a foundational solid-state semiconductor technology widely utilized as a photosensor in a vast array of imaging systems. This technology is extensively employed in scientific research applications where high quantum efficiency and excellent linearity are essential for obtaining quantitatively reliable data. The CCD is implemented as an integrated circuit (IC), meaning that all the necessary components for light detection and signal processing—from the light-sensitive pixels to the transfer mechanisms and readout electronics—are incorporated onto a single, microscopic silicon chip. The operational principle of the CCD relies on the generation, manipulation, and measurement of localized electrical charge packets within the silicon substrate. The process of image capture involves three distinct, sequential phases:

i ***Photon detection and charge accumulation:*** The functional architecture of a CCD is built upon an array of metal–oxide–semiconductor (MOS) capacitors arranged in a grid, which define the individual pixels. When incident photons strike the silicon substrate, they generate electron–hole pairs via the photoelectric effect. The applied potentials within each pixel establish a localized potential well that effectively collects and stores the signal electrons. Crucially, the amount of accumulated charge is directly proportional to the incident light intensity, which is an inherent property known as photometric linearity. This phase concludes after a defined integration period, which is often termed the exposure time.
ii ***Charge transfer (shift register operation)***: Following the integration period, the accumulated charge must be read out. The “coupled” aspect of the CCD design facilitates this essential transfer of charge. A synchronized, multi-phase clocking voltage sequence is applied to adjacent gates along the pixel array. This dynamic manipulation of potential wells effectively shifts the entire packet of accumulated charge, pixel by pixel, first down vertical shift registers and subsequently through a single horizontal shift register. This process occurs without significant loss of charge to maintain image fidelity (high charge transfer efficiency, or CTE).
iii ***Signal readout and analog-to-digital (ADC) conversion:*** The sequenced charge packets are delivered one at a time to a specialized floating diffusion node, which serves as the output node. This node performs the crucial conversion of the discrete charge packet into a measurable analog voltage signal. An on-chip pre-amplifier then boosts this small voltage signal for subsequent processing. At this point in the signal chain, a fixed DC voltage known as **the bias offset** is also intentionally applied to the analog signal to ensure that all subsequent signal and noise values remain positive, thereby preventing negative noise fluctuations from being clipped to zero during analog-to-digital conversion. Within the same amplification and conditioning stage, the primary source of random electronic uncertainty, known as **readout noise (read noise)**, arises. This noise originates mainly from two sources within the amplifier circuitry: thermal noise (or Johnson–Nyquist noise), which arises from the random thermal motion of electrons within the amplifier’s resistors and varies with temperature and bandwidth; and flicker noise (or 1/f noise), which is characterized by power that is inversely proportional to frequency and originates from imperfections in the semiconductor materials. Finally, an external or on-chip analog-to-digital converter (ADC) quantizes the amplified analog signal into discrete digital values, which represent the pixel brightness levels and collectively form the digital image file.

CCD cameras are classified on the basis of their illumination method and charge transfer architecture and function, which are each optimized for specific applications.

*By Illumination Method:*

– **Front-Illuminated CCDs:** Standard designs where light must pass through opaque wiring layers, resulting in lower QE of around 40–60%.
– **Back-Illuminated (biCCD):** The silicon sensor is inverted, allowing light to enter directly from the rear, bypassing internal wiring and achieving high QE (up to 97%) and superior sensitivity for low-light applications.

*By Charge Transfer Architecture and Function:*

– **Full-frame CCDs:** These architectures utilize the entire sensor area for light collection, offering maximal light collection efficiency per unit area. They require the image data to be read out sequentially row by row from the photosensitive area. This slow process results in very low readout noise due to longer integration times, making them suitable for static or long-exposure imaging.
**Interline-transfer/Frame-transfer CCDs:** These designs are optimized for speed and continuous imaging by employing internal mechanisms for rapid charge relocation.
  – *Interline-transfer models* incorporate masked (light-shielded) vertical columns adjacent to every photosensitive pixel column. After a brief exposure, the charge from the illuminated pixels is rapidly shifted sideways into these shielded columns within microseconds. The next exposure can then begin immediately in the active area while the previously stored data are read out.
  – *Frame-transfer models* partition the sensor into two equal halves: an active imaging area and a light-shielded storage array. The entire image is shifted from the imaging area to the storage area in milliseconds. This allows the imaging array to start the subsequent exposure immediately, while the data are read out from the shielded storage array.
  Both mechanisms enable fast electronic shuttering and readout rates suitable for live-cell imaging without motion blur or the need for a mechanical shutter. Faster readout speeds often correlate with slightly higher readout noise compared to full-frame CCDs.
– **Electron-Multiplying CCDs (EM-CCDs):** This functional variation incorporates an on-chip gain register that amplifies weak signals before they encounter the readout electronics. This electronic gain effectively masks the conventional readout noise floor, making it negligible (sub-electron). This unique capability provides several benefits: it enables true single-photon detection, facilitates high frame rates in photon-starved scenarios by allowing very short exposure times, and maximizes the signal-to-noise ratio even at the lowest light levels.

Although the Charge-Coupled Device (CCD) has long been a standard in quantitative research, **Complementary Metal–Oxide–Semiconductor (CMOS)** technology has emerged as a superior alternative for the majority of modern biological imaging applications. The inherent design of CMOS sensors allows them to overcome several key limitations of CCDs, primarily read-out speed and power consumption. CMOS image sensors operate using an active-pixel design, where each pixel integrates its own amplification and readout circuitry. This fundamental architectural difference enables highly parallel data acquisition, contrasting sharply with the serial, bucket-brigade style of charge transfer utilized by CCDs. The classification of CMOS includes the following:

– **Front-Illuminated CMOS or sCMOS:** Similar to CCDs, it is CMOS or sCMOS sensors with conventional designs that route light through opaque wiring layers, providing a low QE value of around 40–60%.
– **Back-Illuminated (bi-sCMOS):** The sCMOS sensor is inverted in back-illuminated designs, allowing light to enter directly from the rear and bypass internal wiring. This architectural change achieves high quantum efficiency (QE) (up to 95%+) and superior sensitivity, which are essential for low-light biological imaging.
– **Scientific CMOS (sCMOS):** To meet the rigorous demands of scientific research, sCMOS (scientific CMOS) technology was developed. The sCMOS dual-amplifier design is an architectural innovation that fundamentally solves the traditional trade-off between sensitivity and dynamic range. In conventional systems (including standard CCDs and CMOS), the user must select a single gain setting, forcing a compromise. Gain, in this context, refers to the electronic amplification factor applied to the signal voltage to increase sensitivity or accommodate brighter signals, which are typically measured as electrons per analog-to-digital unit ($e^{-}$/ADU). Maximizing sensitivity requires high electronic gain to lift dim signals above the read noise floor, but high gain quickly causes bright signals to saturate the sensor, drastically limiting the dynamic range (defined as the ratio between the brightest non-saturating signal and the noise floor). Conversely, low gain accommodates bright signals but buries dim signals in the noise. The sCMOS architecture resolves this issue by capturing both scenarios simultaneously by employing specific design innovations, such as a dual-amplifier design within the column readout structure. This feature allows the sensor to simultaneously read a pixel’s charge through both a high-gain channel and a low-gain channel. These two independent channels function in a complementary manner: the high-gain amplifier channel is optimized for sensitivity and low noise detection, making it ideal for capturing weak fluorescence signals, while the low-gain (high-capacity) amplifier channel is optimized for a large charge capacity or full well depth, allowing it to accurately measure very bright signals without saturation. The data from these two channels are digitized independently and then combined in a sophisticated process executed by the camera’s internal firmware. This merging algorithm typically compares the raw data from both channels pixel by pixel. For pixels where the high-gain channel data are valid (i.e., not saturated), that low-noise value is selected. For pixels that saturated the high-gain channel, the data from the low-gain channel are used. A linear scaling factor is applied to match the values from both channels precisely, producing a single, seamless, high bit-depth image that optimally utilizes both the minimum noise floor and maximum well capacity simultaneously.

The benefits of CMOS technology for bioimaging are significant, offering superior performance across several key metrics. The parallel readout architecture allows for dramatically faster frame rates, which is essential for capturing rapid biological dynamics like calcium signaling or voltage changes in live cells. A key strength of modern sCMOS) is its ability to maintain exceptionally low read noise (often <1e RMS) even when operating at very high frame rates (hundreds of frames per second). This contrasts favorably with many CCDs, where faster readout speeds typically introduce significant increases in read noise. Additionally, because the readout mechanism is more efficient, CMOS sensors consume less power and require less extensive cooling than CCDs, simplifying the design of the system and minimizing thermal artifacts. In addition, incorporating dual gain amplifiers within each pixel enables the measurement of both dim and bright areas simultaneously, capturing a wide range of light intensity in a single image.

Despite these advances, modern CMOS technology still possess some drawbacks compared to the best EM-CCDs. Due to the independent amplification at each pixel site, CMOS sensors typically exhibit higher fixed pattern noise (minor pixel-to-pixel nonuniformity) that requires careful flat-field correction during image processing. Additionally, while high-end sCMOS sensors offer excellent quantum efficiency, the very best scientific EM-CCDs (particularly back-illuminated models optimized for deep cooling) can still achieve slightly superior quantum efficiency (up to 97%) and linearity in extremely photon-starved, long-exposure scenarios. For this reason, EM-CCD remains the superior detector for applications where light is extremely scarce. Examples of several commercially available cameras and their main characteristics are provided in Table 4.

**Bias offset subtraction and strategies for mitigation of read noise.** For quantitative accuracy, the systematic bias offset is calibrated and subtracted using dark frames, which removes the camera’s fixed baseline signal. In bioimaging practice, the camera bias offset often is accounted for, together with the systematic offset that is due to the dark current, by subtracting a master dark frame as described in the previous Section 5.1. In standard sCMOS cameras, the read noise value (typically around 1 electron when expressed as the Root Mean Square (RMS) value) is generated at many parallel output nodes, resulting in minor pixel-to-pixel variability (fixed pattern noise) that requires careful flat-field correction. In contrast, EM-CCDs use a single output node, which, combined with the electron multiplication process, results in a more uniform noise profile and an effective sub-electron read noise floor. The choice of camera is determined by the imaging regime, which is dictated by the signal level relative to the noise floor. In the read-noise limited regime, the signal level (number of incident photons) is so low that the camera’s internal read noise RMS value dominates the total noise profile. Here, the EM-CCD has a distinct advantage: its on-chip gain amplifies the signal above the read noise RMS value, effectively rendering that noise negligible and allowing for the detection of single photons with a high signal-to-noise ratio (SNR). This sensitivity is paramount for highly photon-starved applications such as single-molecule dynamics or imaging radiation-sensitive samples with minimal excitation light.

### 5.3. Light Source Fluctuations

Light source fluctuations are undesirable temporal variations in the intensity of the excitation light. These fluctuations represent a critical source of device-related noise because the measured fluorescence intensity is directly proportional to the intensity of the incident light. As a result, any variations in the light source intensity lead to corresponding changes in fluorescence intensity that can affect the target signal.

**Hardware-based mitigation of light source fluctuations.** The most fundamental and effective approach for minimizing light intensity fluctuations is the employment of inherently stable light sources, such as digitally regulated light-emitting diodes (LEDs) or solid-state lasers, which outperform traditional arc lamps in both temporal stability and long-term reproducibility [134,135]. At the same time, it is important to recognize that many industrial LED illuminators achieve intensity control by pulse-width modulation (PWM) of the drive current [136], and such modulation can make them unsuitable for recording very fast fluorescence signals (<2 ms), whose durations become comparable to the PWM period. In many high-end microscopy systems, stability is further improved through active feedback control, in which a reference photodiode continuously monitors a small fraction of the excitation beam and feeds this signal back to an electronic controller [137,138]. If the measured intensity deviates from the setpoint, the controller dynamically adjusts the drive current to the light source, thereby maintaining nearly constant optical output and substantially reducing long-term drift and slow thermal fluctuations. Below, we provide a brief comparison of common light sources for fluorescence imaging, focusing on stability, lifetime, and spectral characteristics (see also Table 5 for details).

– **Mercury arc lamps** provide broad and intense spectral output with strong emission lines in the UV and visible range that are well suited for widefield fluorescence excitation. However, they exhibit characteristic drawbacks, including arc wander and plasma instabilities that cause flicker and short-term intensity fluctuations, as well as a progressive decrease in output as the lamp ages and the arc envelope degrades. These effects lead to poorer temporal stability and long-term reproducibility.
– **Xenon arc lamps** exhibit a relatively smooth, quasi-continuous spectrum across the visible range and provide more uniform spectral brightness than mercury lamps, which are dominated by discrete emission lines. However, they still suffer from arc wander and plasma instabilities that produce measurable intensity fluctuations, require warm-up, and have a finite lamp lifetime on the order of a few hundred to roughly a thousand hours, leading to gradual output degradation over time.
– **Light-emitting diodes (LEDs)** provide highly stable illumination, with low short-term intensity noise, modest heat generation at the sample plane, and fast, linear electronic modulation, which makes them well suited for time-resolved, quantitative, and ratiometric imaging. Their output is largely free of arc-related flicker seen in discharge lamps, although small long-term drifts can still occur due to temperature-dependent changes in LED junction characteristics and driver electronics, especially in inadequately cooled or unregulated systems [134].
– **Solid-state lasers** provide very high temporal stability and spatial coherence, especially when combined with electronic current regulation and, in high-end systems, active power stabilization based on feedback from a reference photodiode. Their output consists of narrow spectral lines with minimal temporal variation, which makes them particularly well suited for confocal, multiphoton, and super-resolution modalities that require intense, precisely defined, and highly stable excitation beams.

It should be noted that even highly stable light sources exhibit short-term drift as their optical and electronic components reach thermal equilibrium after switching on, which can cause measurable changes in output intensity and spectral characteristics during the first minutes of operation. Therefore, it is recommended to allow a sufficient warm-up period before quantitative imaging so that the illumination source stabilizes at steady-state operating conditions [139].

**Sensor selection**. An efficient solution to cancel out the signal fluctuations caused by the variation in the intensity of the excitation light is to employ sensors for ratiometric measurements [140]. In this technique, two fluorescence signals are simultaneously recorded from the same sensor, and the change in the ratio of these two signals is proportional to the change in the value of the measured property, e.g., membrane voltage or calcium concentration (see Section 2). Because both fluorescence signals change synchronously in response to fluctuations in incident light, the ratio of these signals can be used to eliminate the instability of the light source.

Ratiometric correction can also be performed even if the employed sensor is not ratiometric. This can be accomplished by adding a separate reference fluorophore that is excited by the same light but whose fluorescence does not change during the biological event of interest [141].

### 5.4. Scanning System Noise

In fluorescence microscopy that employs scanning modalities such as confocal and two-photon imaging, galvanometer or resonant mirrors steer the excitation beam across the sample and can introduce several characteristic distortions [132,142,143]. Instability or imperfections in the mechanics and control electronics of these mirrors cause small, random variations in the beam position, which appear as image jitter and frame-to-frame displacements [144]. At high scan rates, the intrinsically nonlinear motion of resonant or galvanometer scanners can also produce nonuniform pixel spacing and geometric distortions or stretching along the fast and slow scan axes [145], while synchronization errors between the mirror position (which defines pixel location) and detector readout timing (which records photons) give rise to systematic line- or frame-periodic artifacts across the image [146,147].

Beyond the noise sources inherent to the scanning system, the effects of the environment must be taken into account. Vibrational noise from sources such as traffic, HVAC systems, or other lab equipment can be transmitted to the microscope stage and scanning components, increasing mirror instability [99,148,149,150]. This environmental noise results in a misalignment of image features, blurring, and spurious signal fluctuations.

**Hardware-based scanning system noise mitigation.** Addressing noise in scanning systems involves specific hardware solutions. One effective method is the employment of systems equipped with rapid and precise electronic feedback control. In advanced galvanometer scanners, the mirror’s position is constantly monitored against its target [147,151,152]. Any discrepancies are immediately corrected by adjusting the motor’s current, which significantly minimizes random jitter. Another widely applicable strategy is signal averaging during acquisition, which is achieved by repeatedly scanning the same line or frame. This technique reduces random noise components, including mirror jitter, improving image clarity [143,153]. To prevent external vibrational noise, it is essential to place the microscope on a pneumatic isolation table [150]. This serves as the primary defense against low-frequency external vibrations that could otherwise induce translational or rotational jitter in the mirror system.

**Post-processing scanning system noise mitigation.** Scanning system noise caused by mirror jitter and external vibration can be mitigated by motion correction algorithms. For vibrational noise or global low-frequency scanner drift, a rigid registration method can be utilized [154,155]. The algorithm selects a stable reference frame (often the mean or the first image) and determines the global translational and rotational shifts of every subsequent frame by maximizing the cross-correlation between the current frame and the reference. Then, each frame is aligned with the reference image. When more complex motion artifacts appear, such as those originating from nonlinear scanner movement, the rigid model is insufficient. Instead, the image is divided by a fine grid into small pieces, and each piece is corrected employing non-rigid registration algorithms [156,157].

**Device-related noise in voltage vs. calcium imaging.** Camera noise (read noise and dark noise) is generally more significant for voltage imaging than for calcium imaging. This difference stems from several factors, including the lower concentration of voltage indicators in the target region, the differences in sensor intensity and/or sensitivity, and the inherently faster kinetics and smaller amplitudes of the recorded voltage signals. Both techniques are susceptible to fluctuations in the light source. Any instability in excitation intensity is directly encoded as a false fluorescence signal change, necessitating hardware stabilization and/or the employment of ratiometric sensors. Scanning system noise becomes more critical at faster acquisition rates and, consequently, requires more attention for high-temporal-resolution imaging that utilizes voltage indicators.

## 6. Sample-Related Measurement Errors

### 6.1. Tissue Scattering

Scattering affects the target fluorescence signal in two ways. It reduces the signal by decreasing the number of both the excitation photons reaching the fluorophore and the photons emitted by the fluorophore reaching the detector. In addition, photons scattered from out-of-focus regions contribute to the recorded fluorescence signal. Strategies for mitigating scattering noise focus on both reducing scattering and eliminating interfering scattered light.

**Hardware-based strategies for reducing scattering-induced measurement errors**. The most efficient strategy to decrease the measurement error due to tissue scattering is to use longer excitation light wavelengths, as the magnitude of Rayleigh scattering is inversely proportional to the fourth power of the wavelength. The employment of two- and three-photon microscopy (2P-M, 3P-M) shifts the excitation wavelengths from the visible range (λ≈ 450 nm to 700 nm) utilized by single-photon techniques into the near-infrared range (λ≈ 700 nm to more than 1700 nm) [158,159,160]. This shift significantly minimizes scattering, enabling deeper tissue penetration.

A further benefit of multiphoton microscopy, in contrast to single-photon techniques, is the reduced excitation of fluorophores outside the specified region of interest. With single-photon confocal microscopy, this reduction can be accomplished only by decreasing the size of the camera pinhole.

Despite the advantages of multiphoton microscopy, the application of this technique is subject to several limitations. In practice, although two-photon laser-scanning microscopes use essentially the same scanners and detectors as conventional confocal systems and can achieve comparable line-scan speeds, they are often operated at lower effective frame rates because of constraints such as the required excitation power, signal levels, and the need to limit photodamage in deep tissue imaging. Furthermore, the reduced photon counts per fluorophore, due to the less efficient nonlinear excitation process, result in a low photon yield and a greater impact from photon shot noise [100].

**Sensor selection.** A possible strategy to decrease tissue scattering is to select sensors with absorption and/or emission spectral bands shifted to the longer wavelength region.

**Voltage vs. calcium imaging.** Tissue scattering has a more significant impact on voltage imaging than on calcium imaging, which is primarily due to the considerable disparity in the magnitudes of the target signals. Scattering attenuates the target signal and introduces a haze, which readily obscures the inherently faint voltage-induced signal. In contrast, the robustly amplified calcium signal demonstrates greater resilience to these effects.

Major research efforts have focused on developing sensors with a red-shifted absorption spectrum. This has resulted in a toolkit of voltage- and calcium-sensitive dyes, GEVIs, and GECIs with absorption maxima exceeding 600 nm [26,68,69,70,93,118,161,162,163,164,165]. The application of two-photon imaging is standard practice for in vivo functional imaging, which is utilized for voltage and calcium sensors [126,166,167,168,169,170]. However, sensors that perform robustly under single-photon illumination can occasionally exhibit poorer and more variable results in two-photon microscopy measurements [171].

### 6.2. Autofluorescence

Autofluorescence is another significant source of background interference in biological fluorescence microscopy. This intrinsic fluorescence originates from natural cellular constituents, such as metabolic cofactors such as NADH and flavins, and structural components such as lipofuscin, collagen, and elastin [172]. The strongest autofluorescence signal is observed in the blue and green regions of the spectrum. Another detrimental effect of autofluorescence is the amplification of noise that increases with the number of photons detected by the detector, i.e., photon shot noise.

**Hardware-based strategies to reduce measurement errors caused by autofluorescence.** A common practical solution to mitigate autofluorescence is to use emission bandpass filters designed to transmit only the narrow band of wavelengths corresponding to the emission band peak of the sensor and to block other wavelengths corresponding to autofluorescence [173]. However, this method is most effective only when the sensor’s emission band has minimal spectral overlap with the tissue autofluorescence. Another strategy is to use excitation light in the red region or to employ multiphoton microscopy with excitation wavelengths shifted to the optical transparency window of biological tissues [174,175]. Finally, autofluorescence can be reduced by confining the illumination to a restricted region of the site of interest or by blocking the autofluorescence from the out-of-focus area.

**Post-processing correction of autofluorescence.** Autofluorescence can be separated from the desired fluorescence signal when imaging is performed with microscopes equipped with spectral detectors that record the full emission spectrum at each pixel. In this case, spectral unmixing algorithms [176,177,178] can be used to estimate and subtract the autofluorescence contribution from the recorded signal.

**Sensor selection.** Given that the autofluorescence signal is most prominent in the blue and green spectral regions (from ∼400 to 550 nm), the optimal strategy is to utilize excitation wavelengths in the far-red region. This strategy implies choosing the sensors with the most red-shifted absorption band from the available options. For this reason, the development of red-shifted absorption band sensors [26,68,69,70,93,118,164] is especially crucial for advances in the fields of biological voltage and calcium imaging. Furthermore, since autofluorescence increases the impact of photon shot noise, the selection of brighter and/or more sensitive sensors is preferable for the reasons described in Section 4.

**Voltage vs. calcium imaging.** Autofluorescence is an important interference factor for the majority of the available voltage and calcium sensors, as they have the highest absorption and emission in the blue and green regions of the spectrum [30,57,179,180,181]. Autofluorescence interference is more critical when using voltage indicators as a result of the significantly smaller amplitudes of the voltage-induced fluorescence signal compared to the high amplitude calcium-induced signal.

### 6.3. Motion Artifacts

Motion artifacts represent a significant source of error in live-cell and in vivo measurements, originating from the relative movement of the biological sample with respect to the microscope objective. In the context of live animal imaging, these artifacts are typically attributed to physiological processes such as cardiac pulsation and respiration as well as general animal locomotion.

**Hardware-based strategies to remove motion artifacts.** Since motion artifacts change the local concentration of voltage indicators at the focal volume, switching to a concentration-independent property such as fluorescence lifetime is a viable solution. Fluorescence Lifetime Imaging Microscopy (FLIM) reports the excited-state lifetime of the indicator, which is largely independent of fluorophore concentration and moderate excitation intensity fluctuations [182,183]. As a result, FLIM-based measurements can, in principle, distinguish voltage- or calcium-dependent lifetime changes from intensity fluctuations caused by sample motion. However, despite all its advantages, FLIM also faces challenges, which are mainly due to the slower scanning speeds required to collect enough photons for statistical accuracy [184].

**Post-processing treatment.** Motion correction algorithms, previously discussed in Section 5.4 in the context of mitigating scanning system noise, are also employed to remove motion artifacts. Both rigid motion correction algorithms, which align each frame with the stable reference frame, and non-rigid motion correction algorithms, which consider each frame as a grid of control points, can be employed [154,155,156,157].

**Sensor selection.** Motion artifacts manifest themselves as changes in fluorescence intensity, primarily resulting from alterations in the concentration of fluorophores within the focal volume due to sample movement. Consequently, ratiometric sensors, which are also effective in mitigating fluorescence oscillations caused by light source fluctuations (as discussed in Section 5.3), can be used similarly to diminish these motion artifacts [185,186,187].

**Voltage vs. calcium imaging.** Motion artifacts pose a significant challenge for voltage imaging, which is primarily because voltage-induced signals are weaker and are collected from a very small area of the plasma membrane. Consequently, even a subtle sample displacement can lead to the loss of the functional signal, capturing instead an area outside the region of interest. This abrupt shift in fluorescence intensity is registered as a substantial artifactual spike [188] that can overshadow the genuine small voltage transient, thus complicating the accurate measurement of voltage dynamics. Motion artifacts pose a weaker restriction on calcium imaging due to the larger collection area and the stronger fluorescence signal. Consequently, the signal perturbation induced by the movement of the sample is less significant.

### 6.4. Photobleaching

Photobleaching is the irreversible photochemical destruction of a fluorophore molecule by excitation light [189], which manifests itself as the degradation of artificial signals over time. In practice, some fluorescent indicators also display reversible photophysical processes (photoswitching), in which molecules stochastically transition between emissive and non-emissive (or spectrally shifted) states under illumination [190,191].

**Hardware-based solutions.** The fundamental approach to mitigating photobleaching is to decrease the sensor’s cumulative interaction with excitation light while maintaining a sufficient signal for detection. Simply decreasing light intensity is a viable solution, but only up to a point, since a certain minimum illumination threshold is always required to achieve detectable fluorescence. Dimmer sensors inherently require higher illumination intensities, leading directly to faster photobleaching. Alternatively, the duration of light interaction with the sensor can be minimized, e.g., by using episodic imaging protocols that rely on intermittent light exposure rather than continuous illumination [192,193]. In long recordings, the photobleaching-caused loss of signal can be compensated by incrementally increasing the illumination power during the experiment [194,195]. Finally, because photobleaching alters the concentration of active sensors, switching to a concentration-independent property such as fluorescence lifetime is another viable solution [182,183].

**Post-processing.** For data analysis, photobleaching manifests itself as a slow, systematic exponential decay in the fluorescence signal baseline. In most modern experiments, responses are quantified as $\Delta F/F_{0}$, where ΔF is the stimulus-evoked change in fluorescence and $F_{0}$ is the baseline fluorescence. This normalization largely compensates for the gradual loss of fluorescence due to dye bleaching or diffusion and makes traces more comparable across time and between regions. Computational detrending algorithms can be additionally applied to model and subtract residual baseline decay from the raw fluorescence trace, commonly using exponential or spline-based curve fitting, where a decay function is fitted to the data and subtracted [196,197].

**Sensor selection.** Sensor selection strategies for photobleaching noise rely on both sensor choice and measurement methods. Since photostability is one of the key properties of sensors—whether they are dyes or genetically encoded proteins—that is constantly improved during design, selecting the most photostable sensor available is the fundamental way to minimize signal degradation during experiments. In addition to the choice of a sensor with higher photostability, the use of ratiometric sensors offers a critical advantage. If the two fluorescence signals measured from a ratiometric sensor have the same photobleaching rate, their ratio will not be altered by photobleaching and can be used to evaluate the signal of interest (see Section 2) [185,198,199].

**Voltage vs. calcium imaging.** Voltage imaging often operates at higher effective acquisition rates and with smaller fractional fluorescence changes than many population-level calcium-imaging experiments, so in practice, it is frequently performed at higher excitation intensities, especially for dim rhodopsin-based GEVIs. This increased light dose can substantially accelerate photobleaching and elevate the risk of phototoxicity. Consequently, the development of highly photostable dyes and genetically encoded sensors has long been a major research direction [200,201]. Successful advancements include VoltageFluors, a newer class of voltage-sensitive dyes that employ a photoinduced electron transfer mechanism to achieve higher photostability than that of previous generation dyes [164]. Furthermore, several modern archaerhodopsin-3-based GEVIs exhibit relatively slow photobleaching under continuous illumination, but this apparent stability must be weighed against their much lower brightness [71].

### 6.5. Mistargeted Sensors

To accurately report a target physiological process, such as a voltage or calcium change, the sensor must be precisely located within the biological cell. Voltage indicators must be inserted into the plasma membrane or a specific membrane compartment to sense the electric field across the lipid bilayer, whereas calcium indicators must be soluble in the cytosol to measure calcium concentration. Mistargeted sensors—probes that either fail to reach their intended subcellular location or label excitable cells outside the region of interest (ROI)—can substantially degrade data quality by generating non-responsive fluorescence and, in the case of active neighboring cells, additional activity-dependent signals that contaminate the measurement within the ROI. This issue is relevant for both calcium indicators and voltage-sensitive dyes and can be mitigated by restricting dye loading or indicator expression to the cells of interest and by careful ROI selection and masking during analysis.

**Hardware-based solutions.** Similar to autofluorescence, mitigation of the signal from mistargeted sensors can be performed by confining the illuminated area (e.g., employing multiphoton microscopy) or the area from which the photons are collected (e.g., employing a smaller pinhole size in confocal microscopy).

**Post-processing.** Post-processing algorithms designed to mitigate background fluorescence originating from mistargeted sensors in the recorded signal are based on the following observation: the target signal oscillates, following the change in membrane voltage or calcium concentration, while the fluorescence from sensors trapped in non-target regions remains constant over time. Several approaches have been developed to exploit this difference.

– **Standard deviation-based algorithms** identify the functional region of interest through temporal variability by calculating the standard deviation of intensity over time for each pixel [66,202]. Pixels that fluctuate strongly are identified as active signal areas, while pixels with high fluorescence intensity but low temporal fluctuation are considered background and computationally excluded.
– **Pixel correlation algorithms** represent another class of highly effective methods that utilize the fact that pixels belonging to the region of interest are highly correlated in time [203]. When the cell undergoes a voltage or calcium change, all pixels in that functional area rise and fall together coherently. Conversely, pixels containing only random noise or static background fluorescence are uncorrelated with this dynamic activity. Pixel correlation algorithms calculate the temporal cross-correlation coefficient between pixel traces; then, correlated pixels are retained, and uncorrelated noise is excluded. This methodology forms the core of many advanced analysis tools, including Constrained Nonnegative Matrix Factorization, which is widely used to demix signals and reliably identify cell footprints in noisy, dense imaging data [204,205,206].

**Sensor selection.** Sensor selection strategies aim to enhance the localization of the fluorescent sensor at the specific site of interest while minimizing the signal from non-target areas. For organic dyes, there are no simple strategies to target the sensor to a specific cell type after bulk application. Researchers attempt to overcome this deficiency by developing fluorogenic dyes that become fluorescent only after the specifically designed caging group is removed by a light stimulus or interaction with a specific enzyme; for a review, see [207]. For genetically encoded sensors, cell-specific promoters are used to drive the expression of the sensor gene exclusively within the desired cell type, providing high specificity. However, even within the target cell, many genetically encoded sensors demonstrate problems with intracellular localization. Mitigation of this problem requires additional genetic manipulations, such as adding trafficking motifs to ensure efficient transport to the subcellular location [208,209,210].

Background fluorescence arising from mistargeted sensors has a larger impact on voltage indicators, as their inherently smaller voltage-dependent signal changes make them more susceptible to contamination by nonspecific fluorescence than calcium indicators. For synthetic dyes, the issue is often less about mistargeting within the cell and more about nonspecific staining across the entire tissue. The solution to this problem requires the employment of strategies for achieving cellular specificity. For instance, photoactivatable dyes can be used. These dyes are bulk-loaded into the sample but remain non-fluorescent until they are locally illuminated at the precise site of interest [211]. Alternatively, enzyme-activatable dyes can be involved [119,207]. This strategy requires preliminary genetic engineering to introduce a specific non-native enzyme into the target cell type. The dye precursor is then applied to the entire sample using a bath application method. Even though the dye precursor enters and stains all cells, it becomes fluorescent only when the caging group is chemically cleaved by the spatially restricted enzyme in the target cell type.

The problem of mistargeted sensors in genetically encoded calcium indicators primarily occurs when the sensor is expressed in organelles, such as the nucleus or mitochondria [37]. In this case, the sensor reports the distinct Ca^2+^ dynamics of that specific organelle rather than the intended bulk cytosolic calcium concentration. The same signal misinterpretation occurs with synthetic calcium-sensitive dyes that can be passively taken up by mitochondria.

For GEVIs, mislocalization presents an even greater challenge because precise localization in the plasma membrane is required to accurately sense voltage dynamics [208,212,213]. When a GEVI protein is misfolded or not trafficked correctly, it can become trapped in the cytosol or form aggregates in the endoplasmic reticulum. These non-functional proteins generate a non-responsive background signal. Researchers are constantly addressing this issue, proposing newer GEVI versions with significantly improved membrane localization [28,32,65,208]. For instance, the inclusion of membrane trafficking motifs in archaerhodopsin-3-based GEVIs (e.g., the endoplasmic reticulum export sequence and the Golgi export trafficking signal) has enabled a substantial enhancement of membrane targeting [214,215].

### 6.6. Toxicity and Phototoxicity

Toxicity and phototoxicity can severely limit the quality and physiological relevance of biological sample imaging by damaging cells and tissues. Toxicity refers to the damage caused by the presence of the indicator in the cell in the absence of light. Phototoxicity is the cellular damage caused by the photophysical and photochemical processes occurring after light excitation. The interaction generates highly destructive species, such as reactive oxygen species (ROS) and other radicals [216,217,218], which damage cellular macromolecules, including DNA and lipids, leading to cellular stress and eventual death. It is important to note that damaging reactive species are generated not only by the sensor fluorophores but also by the photodestruction of intrinsic tissue chromophores, such as NADH and tryptophan. This intrinsic damage is especially significant when using high-energy UV light. Whenever possible, aspects of the physiology under study should therefore be compared in preparations with and without the sensor (or with different expression/loading levels) to assess the magnitude of perturbation, bearing in mind that any measurement unavoidably disturbs the system but should do so minimally for the process of interest.

**Hardware-based solutions.** Hardware strategies aim to reduce the phototoxicity and photodamage of biological tissues by reducing the energy of the excitation light. The first approach is to decrease the overall light–fluorophore interaction, which is the same strategy used for photobleaching mitigation: this involves decreasing illumination power, i.e., using the minimum intensity required for detection, or using a temporal confinement of illumination via episodic imaging protocols to minimize the cumulative photon dose [192,193]. A second and more effective strategy involves the use of less damaging light of a longer wavelength, which can be achieved by switching to multiphoton microscopy [217].

**Sensor selection.** Sensor selection strategies focus on minimizing damage by optimizing the probe itself and its concentration within the cell. One of the fundamental strategies to decrease phototoxicity is the selection of more photostable and red-shifted indicators. The toxic effects of indicators can be minimized by careful optimization of the dye concentration [219,220] or the expression level of genetically encoded indicators. For genetically encoded sensors, this means selecting the correct cell-specific promoter and carefully titrating the viral dose to prevent overexpression, which often leads to toxic protein aggregation and saturation of the cell’s native buffering pathways [221]. In addition, some studies employing genetically encoded sensors include “dead sensor” controls—mutant versions of the same indicator that preserve expression, targeting, and photophysics but lack responsiveness to the target variable [4,222,223]. These “dead” sensors help to quantify how much the active sensor perturbs physiology and to distinguish true biological signals from indicator-induced effects.

The problem of toxicity and phototoxicity in voltage and calcium imaging is constantly addressed during sensor development. Modern versions of voltage and calcium sensors are routinely benchmarked against negative effects on cell physiology, and the results are documented in corresponding research articles. Optimal protocols are established by specifying the appropriate concentrations of synthetic dyes [54,220] and selecting effective cell-specific promoters and viral doses for genetically encoded sensors to prevent toxicity induced by overexpression. Furthermore, the issue of photodamage is actively mitigated through the expansion of the red-shifted indicator palette [68,120]. The effectiveness and compatibility of many sensors are also systematically tested using two-photon microscopy techniques [106,170,224,225].

### 6.7. Perturbation of Target Property

Perturbation of the target property refers to the problem when the sensor alters the measured property, such as membrane voltage or intracellular calcium concentration. This intererence is crucial in both voltage and calcium imaging because the indicator is not a passive reporter but an active component of the cellular environment.

**Sensor selection.** An ideal strategy is to use a ‘silent’ sensor that does not alter the measured property. However, since completely silent sensors do not exist, the best option is to use probes that only minimally perturb the system, for example by choosing designs with reduced Ca^2+^ affinity or minimized interactions with native channels and membranes. The expression level (for genetically encoded sensors) or concentration (for synthetic indicators) should also be carefully optimized so that measurements are performed in a regime where additional buffering or membrane loading remains small relative to the native dynamics. In parallel, nonfunctional “dead” sensor variants that preserve expression level, targeting, and photophysics but lack sensitivity to the measured variable can be employed as critical controls to determine the magnitude of sensor perturbation on the property being measured.

**Perturbation of the target property problem in voltage and calcium imaging.** In voltage imaging, the main concern is any alteration of the cell’s electrical properties. Charged voltage-sensitive dyes can substantially increase the total membrane capacitance when they partition into the lipid bilayer, thereby altering native voltage kinetics [55,102]. This issue has been largely mitigated in more recent VSD versions such as VoltageFluors, which were engineered to exert negligible effects on membrane capacitance and intrinsic electrical behavior while retaining high voltage sensitivity [226]. Similarly, early generations of GEVIs could perturb electrophysiology by altering membrane capacitance or generating photocurrents that directly change membrane voltage [66,227], but these issues have been successfully addressed through subsequent sensor optimization.

For calcium sensors, the perturbation manifests itself primarily as interference with Ca^2+^ homeostasis. Synthetic Ca^2+^ dyes and GECIs act as exogenous buffers: by binding Ca^2+^; they can affect the kinetics and amplitude of the native signal [228]. When using small-molecule dyes, a careful optimization of dye concentration is essential to limit additional buffering while still achieving sufficient SNR. Selecting the latest generations of GECIs that have been rationally designed to reduce interference with cellular signaling pathways and minimize buffering is a good strategy to avoid the problem [229].

### 6.8. pH-Sensitivity

The pH sensitivity of several GEVIs represents a significant source of artifact in voltage imaging not as random noise but as a competing physiological signal. Small, localized changes in intracellular or extracellular pH frequently accompany normal neuronal and cardiac activity, and when a GEVI is pH-sensitive, these changes can produce fluorescence variations that are superimposed on, and sometimes mistaken for, genuine voltage-dependent signals [230,231,232]. Moreover, activity-dependent pH shifts can be spatially nonuniform within a cell, leading to compartment-specific baseline changes, which further complicates interpretation [231].

**Post-processing signal treatment.** Because pH-related fluorescence changes typically evolve more slowly (seconds to tens of seconds) than many fast voltage transients, temporal filtering or baseline-drift correction can, in some cases, reduce their impact. However, such approaches must be applied with caution: slow pH drifts are themselves biologically driven, may differ across compartments, and simple high-pass filtering can distort slow components of the true voltage signal or introduce spatially non-uniform artifacts [231].

**Sensor selection.** The simplest solution is to use the GEVI, which does not demonstrate pH sensitivity. Another option is to co-express a spectrally distinct, genetically encoded pH sensor alongside the GEVI. After measuring voltage and pH fluctuations simultaneously, the latter can be mathematically subtracted from the total GEVI signal, isolating the true voltage trace [232].

## 7. Conclusions

To ensure reliable data acquisition, researchers performing quantitative biological imaging with voltage and calcium indicators must meticulously manage the full spectrum of noise sources and other experimental errors. This requires a systematic approach that simultaneously maximizes the efficiency of the imaging hardware and optimizes the experimental methodology through careful sensor selection and sample preparation.

The first imperative is hardware optimization. Camera instrumental errors (e.g., dark current, read noise, and dark noise) can be significantly mitigated through the careful selection and operation of research-grade sCMOS or EE-CCD cameras. Careful selection of the camera and operational recommendations following, such as cooling the detector and optimizing readout speeds, allows researchers to significantly reduce camera-based errors and achieve the shot-noise limited regime. In this optimal state, instrumental noise is negligible, and signal quality is fundamentally constrained only by the unavoidable Poisson statistics of photon detection, which can be managed by well-developed post-processing algorithms (ranging from convenient averaging and filtering to more advanced deep-learning denoising algorithms). The camera offset should also be evaluated and subtracted.

To achieve the desirable shot-noise limited regime, instrumental manipulation to improve photon collection efficiency (e.g., higher numerical aperture (NA) objectives, increased exposure time, and selecting a camera with higher quantum efficiency (QE)) can also be required. These adjustments are particularly critical in several scenarios, such as high-speed voltage imaging, deep-tissue two-photon microscopy, or when dim sensors such as Arch-based GEVIs are involved. In worst case, whan the raw signal lies close to the fundamental noise floor, more complex mathematical models must be employed during the post-processing stage to evaluate the target signal if it is still fundamentally possible.

Instrumental errors arising from microscope components other than the camera (e.g., light source fluctuations and scanning system noise) should also be considered. The illumination source should be carefully selected and thoroughly warmed up before measurement, scanning systems equipped with rapid and precise electronic feedback control should be employed, and the effect of external vibrations should be minimized.

Careful consideration of sample-related error sources is also required from a researcher performing imaging experiments. These error sources encompass those arising from a variety of phenomena, such as tissue scattering, autofluorescence, mistargeted sensors, photobleaching, phototoxicity, and perturbation of the target property. Mitigation strategies often require a careful selection of a sensor with optimal properties, as well as microscopy techniques and post-processing algorithms. As a rule, sensors with red-shifted absorption/emission bands are preferable to reduce tissue scattering, autofluorescence, and phototoxicity. Mistargeting problems are more easily addressed by genetically encoded sensors than by synthetic dyes. The use of ratiometric sensors and the fluorescence lifetime imaging technique (FLIM) offers concentration-independent readouts that help to address the issue of photobleaching and some forms of motion artifacts. Optical sectioning techniques (confocal and multiphoton microscopy) are crucial for addressing mislocalization problems and autofluorescence. The problem of photobleaching can also be addressed by optimizing illumination power, employing episodic imaging protocols, or computational detrending algorithms. Finally, problems caused by motion artifacts can be eliminated by applying motion correction algorithms.

Generally, planning a biological imaging experiment requires careful selection of both sensors and experimental methodologies, precise instrumental adjustments, and a thoughtful choice of post-processing algorithms. The future enhancement of spatiotemporal resolution, stability, and accuracy of measurements in biological imaging in general, and in voltage and calcium imaging in particular, will require advancements across all key areas: the development of more efficient sensors, the improvement of hardware and methodologies, and the implementation of new advanced signal post-processing algorithms.

## Figures and Tables

**Table 1:** Examples of commerically available voltage-sensitive dyes (VSDs) and genetically encoded voltage indicators (GEVIs) and their key optical and voltage-sensitivity parameters.

Sensor	$\lambda_{\mathrm{ex}}$ (nm)	$\lambda_{\mathrm{em}}$ (nm)	FQY (%)	ΔF/F per 100 mV (%)
**Voltage-sensitive dyes (VSDs) **				
Di-4-ANEPPS	468 [20]	617 [21]	30 [21]	10 [21]
CytoVolt1 (Di-4-ANBDQBS)	528 [22]	689 [22]	30 [22]	19 [23]
ANNINE-6plus	488 [24]	600 [24]	n/d	30 [24]
ElecroFluor630 (Di-4-ANEQ(F)PTEA)	547 [21]	686 [21]	10 [21]	12 [21]
VF1.4.Cl	521 [25]	534 [25]	24 [25]	20 [25]
FluoVolt (VF2.1.Cl)	522 [25]	535 [25]	5.7 [25]	27 [25]
BeRST1	658 [26]	683 [26]	2.2 [26]	24 [26]
diEt BeRST	658 [27]	673 [27]	13 [27]	40 [27]
**Genetically encoded voltage indicators (GEVIs)**				
ASAP5	480 [28]	525 [28]	∼70 [29]	−59 [28]
ArcLight-A242	488 [30]	515 [30]	n/d	−35 [30]
QuasAr2	590 [31]	715 [31]	0.4 [31]	90 [31]
Archon1	585 [32]	740 [32]	0.5 [33]	81 [32]
Ace2-mNeon2	506 [34]	518 [34]	∼80 [35]	−26 [34]

**Table 2:** Examples of commerically available calcium-sensitive dyes (CSDs) and genetically encoded calcium indicators (GECIs) and their key optical and sensitivity parameters.

Sensor	$\lambda_{\mathrm{ex}}$ (nm)	$\lambda_{\mathrm{em}}$ (nm)	$K_{d}$ (nM)	Sensitivity
**Calcium-sensitive dyes**				
Rhod-2	550 [36]	580 [36]	500 [37]	>100-fold upon binding [37]
Fluo-4	495 [38]	528 [38]	345 [38]	>100-fold upon binding [38]
Fluo-8	494 [39]	517 [39]	390 [39]	200-fold upon binding [39]
Fura-red	430/480 [36]	630/652 [36]	140 [36]	n/d
**Genetically encoded calcium indicators**				
XCaMP-Gf	490 [40]	515 [40]	154 [40]	25% per AP [40]
jGCaMP7fb	500 [41]	515 [41]	150 [42]	98% per AP [41]
jGCaMP8f	500 [41]	515 [41]	334 [42]	38% per AP [41]
jGCaMP8s	500 [41]	515 [41]	n/d	183% per AP [41]
PinkyCaMP	568 [43]	600 [43]	54 [43]	12.8% per AP [43]

**Table 3:** Summary of noise sources and strategies for improvement of signal-to-noise ratio.

Measurement Error		Mitigation Strategies
**Fundamental noise due to the quantum nature of photons**		
**Photon shot noise** Relevant to all optical measurements; follows the Poisson statistics, so the signal-to-noise ratio increases as the square root of the detected photon count; in the **photon shot-noise limited regime**, which is the best possible regime for high-quality biological measurements, the only instrumental noise source; the SNR is lower when the intensity of the recorded fluorescence signal is lower, i.e., high-speed voltage imaging, two-photon microscopy; it is more significant when dim sensors are employed (archaerhodopsin-3-based sensors)		**Hardware-based SNR enhancement:** Increasing incident light power (can increase photobleaching and temperature of the sample) Employing objectives with higher numerical aperture Increasing exposure time Optimizing pinhole size (confocal microscopy) Employing cameras with higher quantum efficiency (usually back-illuminated versions of CCD and sSMOS) **Post-processing noise mitigation:** Averaging over multiple trials (periodic events) Temporal averaging over consecutive frames Spatial averaging within region of interest Employing low-pass filtering algorithms Employing dimensionality reduction algorithms **Sensor selection:** Employing brighter sensors Employing more sensitive sensors
**Device-related (Instrumental) measurement error**		
**Camera dark noise** Relevant to all imaging measurements; more significant when the intensity of the recorded fluorescence signal is low; can be effectively reduced and the desired photon shot-limited regime established by proper hardware selection and careful operational instructions following		**Hardware-based noise mitigation:** The camera sensor cooling Exposure time optimization **Post-processing noise mitigation (needed only with extremely scarce photon budget when photon-shot noise regime cannot be established:** Dark frame subtraction to remove systematic offset Temporal or spatial averaging Employing low-pass filtering to remove stochastic component
**Camera read noise** Required special attention when the intensity of the recorded fluorescence signal is low because measurements can be moved out of the desired photon shot-noise regime to the read-noise limited regime; can be effectively reduced by proper hardware selection and careful operational instructions following		**Hardware-based noise mitigation:** Employing EM-CCDs cameras Employing sCMOS cameras with low read noise Decreasing readout speeds
**Device-related (Instrumental) measurement errors**		
**Light source fluctuations** Relevant to all biological imaging measurements; more significant when the intensity of the recorded fluorescence signal is low; can be effectively reduced by proper hardware selection and careful operational instructions following		**Hardware-based mitigation:** Employing digitally controlled illumination sources Warming up light source before experiment **Sensor selection:** Employing ratiometric sensors
**Scanning system noise** Relevant to all biological imaging measurements; more significant when the intensity of the recorded fluorescence signal is low; can be effectively reduced by proper hardware selection and careful operational instructions following		**Hardware-based mitigation:** Employing systems with electronic feedback control Performing several measurements and subsequent averaging Employing a pneumatic isolation table for vibration suppression **Post-processing mitigation:** Employing motion correction algorithms
**Sample-related measurement error**		
**Tissue scattering** Relevant to all biological imaging measurements; requires special attention in the following cases: the intensity of the recorded fluorescence signal is low; sensors with excitation and/or emission in blue or green regions are employed; experiments performed on tissues or organisms but not on a single cell		**Hardware-based mitigation:** Employing multiphoton microscopy Optimizing pinhole size (confocal microscopy) **Sensor selection:** Employing sensors with red-shifted absorption and emission spectra
**Autofluorescence** Relevant to all biological imaging measurements; requires special attention in the following cases: the intensity of the recorded fluorescence signal is low; experiments performed on tissues or organisms but not on a single cell; sensors with excitation and/or emission in blue or green regions are employed		**Hardware-based mitigation:** Employing emission bandpass filters Employing multiphoton microscopy Decreasing pinhole size (confocal microscopy) **Post-processing:** Employing spectral unmixing algorithms **Sensor selection:** Employing sensors with red-shifted absorption and emission spectra Employing brighter sensors Employing more sensitive sensors
**Motion artifacts** More significant for experiments on animals than on tissues or on a single cell		**Hardware-based mitigation:** Employing Fluorescence Lifetime Imaging Microscopy (FLIM) **Post-processing mitigation:** Employing motion correction algorithms **Sensor selection:** Employing ratiometric sensors
**Photobleaching** Relevant to all biological imaging measurements; requires special attention in the following cases: the intensity of the recorded fluorescence signal is low; long-term measurements		**Hardware-based mitigation:** Decreasing illumination power Employing episodic imaging protocols Employing Fluorescence Lifetime Imaging Microscopy (FLIM) **Post-processing:** Employing computational detrending algorithms **Sensor selection:** Employing sensors with improved photostability Employing ratiometric sensors
**Mistargeted sensors** Relevant to all biological imaging measurements; synthetic dyes—non-specific staining across the entire tissue; genetically encoded indicators (especially GEVIs) can demonstrate significant mistargeted expression		**Hardware-based mitigation** Employing multiphoton microscopy Optimizing pinhole size (confocal microscopy) **Post-processing:** Employing standard deviation-based algorithms Employing pixel correlation algorithms **Sensor selection and sample preparation:** Employing ratiometric sensors Employing photoactivated dyes Employing enzyme-activated dyes Introducing cell-specific promoters (GEVI and GECI) Introducing trafficking motifs (GEVI)
**Toxicity and phototoxicity** Relevant to all biological imaging measurements; more significant for dyes than for GEVIs and GECIs; more significant when sensors with excitation in blue or green regions are used		**Hardware-based noise mitigation:** Decreasing illumination power Employing multiphoton microscopy **Sensor selection and sample preparation:** Employing sensors with improved photostability Employing sensors with red-shifted absorption and emission spectra Optimizing dye concentration Employing `dead’ sensors
**Perturbation of target property** For voltage imaging, special attention is required for charged VSDs; for calcium imaging, all types of sensors, CSDs and GECIs can perturb calcium homeostasis.		**Sensor selection and sample preparation:** Employing sensors that do not affect target property Optimizing concentration (dyes) Optimizing expression levels (GEVIs and GECIs) Employing `dead’ sensors
pH-sensitivity (GEVIs, confounding signal) Should be considered only if pH-sensitive GEVIs are employed.		**Sensor selection:** Employing pH-insensitive GEVIs Employing a construct of a pH-sensitive GEVI and a spectrally distinct genetically encoded pH sensor

**Table 4:** Examples of commercially available cameras used for voltage and calcium imaging.

Camera (Vendor)	Sensor Type	Peak QE	Read Noise	Array/Pixel Size
Hamamatsu ORCA-Flash4.0 V3	sCMOS	>70% at 500 nm	<1.3 e^−^	2048 × 2048, 6.5 μm
Hamamatsu ORCA-Lightning	sCMOS	>70% at 550 nm	2.0 e^−^	Up to 4608 × 2592, 6.5 μm
Hamamatsu ORCA-Fusion BT	sCMOS (back-illuminated)	∼95% at 550 nm	∼ 1.4 e^−^	2048 × 2048, 6.5 μm
Andor Zyla 4.2 PLUS	sCMOS	82%	0.9 e^−^	2048 × 2048, 6.5 μm
Andor Sona 4.2B-6	sCMOS (back-illuminated)	95%	1.0 e^−^	2048 × 2048, 6.5 μm
Andor iXon Life 888	EMCCD (back-illuminated)	>95%	<1 e^−^	1024 ×1024, 13 μm

**Table 5:** Comparison of common illumination sources used in high-resolution fluorescence microscopy.

Light Source	Typical Stability (Short-Term)	Typical Lifetime (Order of Magnitude)	Spectral Range/Profile
Mercury arc lamp	Poor–moderate; arc wander and plasma instabilities can produce >5–10% intensity variation over time	∼200 h; lifetime strongly dependent on ignition cycles and proper warm-up	Discrete strong lines in the UV/blue/green with weaker continuum from ∼350–700 nm; rich UV output
Xenon arc lamp	Moderate; generally more stable and with less arc drift than mercury lamps, but still on the order of a few percent	∼500–1000 h, depending on power and operating conditions	Broad, quasi-continuous spectrum across the visible range (≈350–800 nm) with some IR contribution
High-power LED	Very good; spatial and temporal stability often an order of magnitude better than arc lamps (<1% variation under regulated drive)	∼10,000–50,000 h to significant degradation, depending on drive current and cooling	Narrow to moderately broad bands (tens of nm), with modules covering UV to NIR; selectable wavelengths
Solid-state laser	Excellent; with temperature- and current-stabilized operation and power feedback, sub-percent intensity fluctuations over long periods are routine	Thousands of hours; depends on laser type (diode, DPSS, fiber) and operating regime	Very narrow spectral lines (≈0.1–1 nm) at discrete wavelengths from UV to NIR

## Data Availability

The original contributions presented in this study are included in the article. Further inquiries can be directed to the corresponding author.

## References

[1] Kulkarni R.U., Miller E.W. (2017). Voltage imaging: Pitfalls and potential. Biochemistry.

[2] Knöpfel T., Song C. (2019). Optical voltage imaging in neurons: Moving from technology development to practical tool. Nat. Rev. Neurosci..

[3] Klier P.E., Roo R., Miller E.W. (2022). Fluorescent indicators for imaging membrane potential of organelles. Curr. Opin. Chem. Biol..

[4] Gest A.M., Sahan A.Z., Zhong Y., Lin W., Mehta S., Zhang J. (2024). Molecular spies in action: Genetically encoded fluorescent biosensors light up cellular signals. Chem. Rev..

[5] Shakibi R., Yazdipour F., Abadijoo H., Manoochehri N., Pouria F.R., Bajooli T., Simaee H., Abdolmaleki P., Khatibi A., Abdolahad M. (2025). From resting potential to dynamics: Advances in membrane voltage indicators and imaging techniques. Q. Rev. Biophys..

[6] Selim N.A., Wojtovich A.P. (2025). Mitochondrial membrane potential and compartmentalized signaling: Calcium, ROS, and beyond. Redox Biol..

[7] Nikolaev D.M., Mironov V.N., Shtyrov A.A., Kvashnin I.D., Mereshchenko A.S., Vasin A.V., Panov M.S., Ryazantsev M.N. (2023). Fluorescence imaging of cell membrane potential: From relative changes to absolute values. Int. J. Mol. Sci..

[8] Spruston N., Jaffe D.B., Williams S.H., Johnston D. (1993). Voltage-and space-clamp errors associated with the measurement of electrotonically remote synaptic events. J. Neurophysiol..

[9] Scanziani M., Häusser M. (2009). Electrophysiology in the age of light. Nature.

[10] Cohen L., Salzberg B., Grinvald A. (1978). Optical methods for monitoring neuron activity. Annu. Rev. Neurosci..

[11] Zhou W.L., Yan P., Wuskell J.P., Loew L.M., Antic S.D. (2007). Intracellular long-wavelength voltage-sensitive dyes for studying the dynamics of action potentials in axons and thin dendrites. J. Neurosci. Methods.

[12] Lippert M.T., Takagaki K., Xu W., Huang X., Wu J.Y. (2007). Methods for voltage-sensitive dye imaging of rat cortical activity with high signal-to-noise ratio. J. Neurophysiol..

[13] Eisner D., Neher E., Taschenberger H., Smith G. (2023). Physiology of intracellular calcium buffering. Physiol. Rev..

[14] Matthews E.A., Dietrich D. (2015). Buffer mobility and the regulation of neuronal calcium domains. Front. Cell. Neurosci..

[15] Zhou X., Belavek K.J., Miller E.W. (2021). Origins of Ca^2+^ imaging with fluorescent indicators. Biochemistry.

[16] Augustine G.J., Santamaria F., Tanaka K. (2003). Local calcium signaling in neurons. Neuron.

[17] Vyshedskiy A., Lin J.W. (2000). Presynaptic Ca^2+^ influx at the inhibitor of the crayfish neuromuscular junction: A photometric study at a high time resolution. J. Neurophysiol..

[18] Tang S., Reddish F., Zhuo Y., Yang J.J. (2015). Fast kinetics of calcium signaling and sensor design. Curr. Opin. Chem. Biol..

[19] Mutoh H., Mishina Y., Gallero-Salas Y., Knöpfel T. (2015). Comparative performance of a genetically-encoded voltage indicator and a blue voltage sensitive dye for large scale cortical voltage imaging. Front. Cell. Neurosci..

[20] Fluhler E., Burnham V.G., Loew L.M. (1985). Spectra, membrane binding, and potentiometric responses of new charge shift probes. Biochemistry.

[21] Yan P., Acker C.D., Biasci V., Judge G., Monroe A., Sacconi L., Loew L.M. (2023). Near-infrared voltage-sensitive dyes based on chromene donor. Proc. Natl. Acad. Sci. USA.

[22] Panama B.K., Costantino A., Nowak M.W., Daddario A.A., Rasmusson R.L., Bett G.C. (2023). Spectral properties of the voltage-sensitive dye di-4-ANBDQBS. Biophys. J..

[23] Warren M., Spitzer K.W., Steadman B.W., Rees T.D., Venable P., Taylor T., Shibayama J., Yan P., Wuskell J.P., Loew L.M. (2010). High-precision recording of the action potential in isolated cardiomyocytes using the near-infrared fluorescent dye di-4-ANBDQBS. Am. J. Physiol. Heart Circ. Physiol..

[24] Fromherz P., Hübener G., Kuhn B., Hinner M.J. (2008). ANNINE-6plus, a voltage-sensitive dye with good solubility, strong membrane binding and high sensitivity. Eur. Biophys. J..

[25] Miller E.W., Lin J.Y., Frady E.P., Steinbach P.A., Kristan W.B., Tsien R.Y. (2012). Optically monitoring voltage in neurons by photo-induced electron transfer through molecular wires. Proc. Natl. Acad. Sci. USA.

[26] Huang Y.L., Walker A.S., Miller E.W. (2015). A photostable silicon rhodamine platform for optical voltage sensing. J. Am. Chem. Soc..

[27] Navarro M.X., Gerstner N.C., Lipman S.M., Dolgonos G.E., Miller E.W. (2024). Improved Sensitivity in a Modified Berkeley Red Sensor of Transmembrane Potential. ACS Chem. Biol..

[28] Hao Y.A., Lee S., Roth R.H., Natale S., Gomez L., Taxidis J., O’Neill P.S., Villette V., Bradley J., Wang Z. (2024). A fast and responsive voltage indicator with enhanced sensitivity for unitary synaptic events. Neuron.

[29] Topell S., Hennecke J., Glockshuber R. (1999). Circularly permuted variants of the green fluorescent protein. FEBS Lett..

[30] Jin L., Han Z., Platisa J., Wooltorton J.R., Cohen L.B., Pieribone V.A. (2012). Single action potentials and subthreshold electrical events imaged in neurons with a fluorescent protein voltage probe. Neuron.

[31] Hochbaum D.R., Zhao Y., Farhi S.L., Klapoetke N., Werley C.A., Kapoor V., Zou P., Kralj J.M., Maclaurin D., Smedemark-Margulies N. (2014). All-optical electrophysiology in mammalian neurons using engineered microbial rhodopsins. Nat. Methods.

[32] Piatkevich K.D., Jung E.E., Straub C., Linghu C., Park D., Suk H.J., Hochbaum D.R., Goodwin D., Pnevmatikakis E., Pak N. (2018). A robotic multidimensional directed evolution approach applied to fluorescent voltage reporters. Nat. Chem. Biol..

[33] Silapetere A., Hwang S., Hontani Y., Fernandez Lahore R.G., Balke J., Escobar F.V., Tros M., Konold P.E., Matis R., Croce R. (2022). QuasAr Odyssey: The origin of fluorescence and its voltage sensitivity in microbial rhodopsins. Nat. Commun..

[34] Kannan M., Vasan G., Haziza S., Huang C., Chrapkiewicz R., Luo J., Cardin J.A., Schnitzer M.J., Pieribone V.A. (2022). Dual-polarity voltage imaging of the concurrent dynamics of multiple neuron types. Science.

[35] Steiert F., Petrov E.P., Schultz P., Schwille P., Weidemann T. (2018). Photophysical behavior of mNeonGreen, an evolutionarily distant green fluorescent protein. Biophys. J..

[36] Sheng C.Q., Wu S.S., Cheng Y.K., Wu Y., Li Y.M. (2024). Comprehensive review of indicators and techniques for optical mapping of intracellular calcium ions. Cereb. Cortex.

[37] Del Nido P.J., Glynn P., Buenaventura P., Salama G., Koretsky A.P. (1998). Fluorescence measurement of calcium transients in perfused rabbit heart using rhod 2. Am. J. Physiol. Heart Circ. Physiol..

[38] Gee K.R., Brown K., Chen W.U., Bishop-Stewart J., Gray D., Johnson I. (2000). Chemical and physiological characterization of fluo-4 Ca^2+^-indicator dyes. Cell Calc..

[39] Lock J.T., Parker I., Smith I.F. (2015). A comparison of fluorescent Ca^2+^ indicators for imaging local Ca^2+^ signals in cultured cells. Cell Calc..

[40] Inoue M., Takeuchi A., Manita S., Horigane S.i., Sakamoto M., Kawakami R., Yamaguchi K., Otomo K., Yokoyama H., Kim R. (2019). Rational engineering of XCaMPs, a multicolor GECI suite for in vivo imaging of complex brain circuit dynamics. Cell.

[41] Zhang Y., Rózsa M., Liang Y., Bushey D., Wei Z., Zheng J., Reep D., Broussard G.J., Tsang A., Tsegaye G. (2023). Fast and sensitive GCaMP calcium indicators for imaging neural populations. Nature.

[42] Sakamoto M., Yokoyama T. (2025). Probing neuronal activity with genetically encoded calcium and voltage fluorescent indicators. Neurosci. Res..

[43] Fink R., Imai S., Gockel N., Lauer G., Renken K., Wietek J., Lamothe-Molina P.J., Fuhrmann F., Mittag M., Ziebarth T. (2025). PinkyCaMP a mScarlet-based calcium sensor with exceptional brightness, photostability, and multiplexing capabilities. bioRxiv.

[44] Koldenkova V.P., Nagai T. (2013). Genetically encoded Ca^2+^ indicators: Properties and evaluation. Biochim. Biophys. Acta—Mol. Cell Res..

[45] Mertes N., Busch M., Huppertz M.C., Hacker C.N., Wilhelm J., Gürth C.M., Kühn S., Hiblot J., Koch B., Johnsson K. (2022). Fluorescent and bioluminescent calcium indicators with tuneable colors and affinities. J. Am. Chem. Soc..

[46] Paredes R.M., Etzler J.C., Watts L.T., Zheng W., Lechleiter J.D. (2008). Chemical calcium indicators. Methods.

[47] Bullen A., Saggau P. (1999). Optical recording from individual neurons in culture. Modern Techniques in Neuroscience Research.

[48] Lopez-Izquierdo A., Warren M., Riedel M., Cho S., Lai S., Lux R.L., Spitzer K.W., Benjamin I.J., Tristani-Firouzi M., Jou C.J. (2014). A near-infrared fluorescent voltage-sensitive dye allows for moderate-throughput electrophysiological analyses of human induced pluripotent stem cell-derived cardiomyocytes. Am. J. Physiol.-Heart Circ. Physiol..

[49] Miller E.W. (2016). Small molecule fluorescent voltage indicators for studying membrane potential. Curr. Opin. Chem. Biol..

[50] Loew L.M. (2015). Design and use of organic voltage sensitive dyes. Membrane Potential Imaging in the Nervous System and Heart.

[51] Bradley J., Luo R., Otis T.S., DiGregorio D.A. (2009). Submillisecond optical reporting of membrane potential in situ using a neuronal tracer dye. J. Neurosci..

[52] Gonzalez J.E., Tsien R.Y. (1997). Improved indicators of cell membrane potential that use fluorescence resonance energy transfer. Chem. Biol..

[53] Rowland C.E., Brown C.W., Medintz I.L., Delehanty J.B. (2015). Intracellular FRET-based probes: A review. Methods Appl. Fluores..

[54] Chanda B., Blunck R., Faria L.C., Schweizer F.E., Mody I., Bezanilla F. (2005). A hybrid approach to measuring electrical activity in genetically specified neurons. Nat. Neurosci..

[55] Ma Y., Bayguinov P.O., Jackson M.B. (2019). Optical studies of action potential dynamics with hVOS probes. Curr. Opin. Biomed. Eng..

[56] Woodford C.R., Frady E.P., Smith R.S., Morey B., Canzi G., Palida S.F., Araneda R.C., Kristan W.B., Kubiak C.P., Miller E.W. (2015). Improved PeT molecules for optically sensing voltage in neurons. J. Am. Chem. Soc..

[57] Lu X., Wang Y., Liu Z., Gou Y., Jaeger D., St-Pierre F. (2023). Widefield imaging of rapid pan-cortical voltage dynamics with an indicator evolved for one-photon microscopy. Nat. Commun..

[58] Platisa J., Vasan G., Yang A., Pieribone V.A. (2017). Directed evolution of key residues in fluorescent protein inverses the polarity of voltage sensitivity in the genetically encoded indicator ArcLight. ACS Chem. Neurosci..

[59] Abdelfattah A.S., Farhi S.L., Zhao Y., Brinks D., Zou P., Ruangkittisakul A., Platisa J., Pieribone V.A., Ballanyi K., Cohen A.E. (2016). A bright and fast red fluorescent protein voltage indicator that reports neuronal activity in organotypic brain slices. J. Neurosci..

[60] Tsutsui H., Jinno Y., Tomita A., Niino Y., Yamada Y., Mikoshiba K., Miyawaki A., Okamura Y. (2013). Improved detection of electrical activity with a voltage probe based on a voltage-sensing phosphatase. J. Physiol..

[61] Akemann W., Mutoh H., Perron A., Park Y.K., Iwamoto Y., Knöpfel T. (2012). Imaging neural circuit dynamics with a voltage-sensitive fluorescent protein. J. Neurophysiol..

[62] Leong L.M., Kang B.E., Baker B.J. (2021). Improving the flexibility of genetically encoded voltage indicators via intermolecular FRET. Biophys. J..

[63] Zhang X.M., Yokoyama T., Sakamoto M. (2021). Imaging voltage with microbial rhodopsins. Front. Mol. Biosci..

[64] Gong Y., Wagner M.J., Zhong Li J., Schnitzer M.J. (2014). Imaging neural spiking in brain tissue using FRET-opsin protein voltage sensors. Nat. Commun..

[65] Tian H., Davis H.C., Wong-Campos J.D., Park P., Fan L.Z., Gmeiner B., Begum S., Werley C.A., Borja G.B., Upadhyay H. (2023). Video-based pooled screening yields improved far-red genetically encoded voltage indicators. Nat. Methods.

[66] Kralj J.M., Douglass A.D., Hochbaum D.R., Maclaurin D., Cohen A.E. (2012). Optical recording of action potentials in mammalian neurons using a microbial rhodopsin. Nat. Methods.

[67] Nikolaev D.M., Shtyrov A.A., Vyazmin S.Y., Vasin A.V., Panov M.S., Ryazantsev M.N. (2023). Fluorescence of the retinal chromophore in microbial and animal rhodopsins. Int. J. Mol. Sci..

[68] McIsaac R.S., Engqvist M.K., Wannier T., Rosenthal A.Z., Herwig L., Flytzanis N.C., Imasheva E.S., Lanyi J.K., Balashov S.P., Gradinaru V. (2014). Directed evolution of a far-red fluorescent rhodopsin. Proc. Natl. Acad. Sci. USA.

[69] Nikolaev D.M., Mironov V.N., Metelkina E.M., Shtyrov A.A., Mereshchenko A.S., Demidov N.A., Vyazmin S.Y., Tennikova T.B., Moskalenko S.E., Bondarev S.A. (2024). Rational Design of Far-Red Archaerhodopsin-3-Based Fluorescent Genetically Encoded Voltage Indicators: From Elucidation of the Fluorescence Mechanism in Archers to Novel Red-Shifted Variants. ACS Phys. Chem. Au.

[70] Nikolaev D.M., Metelkina E.M., Domskii N.A., Osadchy I.R., Mereshchenko A.S., Bondarev S.A., Zhouravleva G.A., Shtyrov A.A., Panov M.S., Ryazantsev M.N. (2025). Semirational Protein Engineering Yields Archaerhodopsin-3-Based Fluorescent Genetically Encoded Voltage Indicators with Enhanced Brightness and Red Shifted Absorption Bands. Chem. Bio Eng.

[71] Bando Y., Sakamoto M., Kim S., Ayzenshtat I., Yuste R. (2019). Comparative evaluation of genetically encoded voltage indicators. Cell Rep..

[72] Rhee J.K., Leong L.M., Mukim M.S.I., Kang B.E., Lee S., Bilbao-Broch L., Baker B.J. (2020). Biophysical parameters of GEVIs: Considerations for imaging voltage. Biophys. J..

[73] Kannan M., Vasan G., Huang C., Haziza S., Li J.Z., Inan H., Schnitzer M.J., Pieribone V.A. (2018). Fast, in vivo voltage imaging using a red fluorescent indicator. Nat. Methods.

[74] Gong Y., Huang C., Li J.Z., Grewe B.F., Zhang Y., Eismann S., Schnitzer M.J. (2015). High-speed recording of neural spikes in awake mice and flies with a fluorescent voltage sensor. Science.

[75] Abdelfattah A.S., Valenti R., Zheng J., Wong A., Podgorski K., Koyama M., Kim D.S., Schreiter E.R. (2020). A general approach to engineer positive-going eFRET voltage indicators. Nat. Commun..

[76] Inagaki S., Tsutsui H., Suzuki K., Agetsuma M., Arai Y., Jinno Y., Bai G., Daniels M.J., Okamura Y., Matsuda T. (2017). Genetically encoded bioluminescent voltage indicator for multi-purpose use in wide range of bioimaging. Sci. Rep..

[77] Srinivasan P., Griffin N.M., Thakur D., Joshi P., Nguyen-Le A., McCotter S., Jain A., Saeidi M., Kulkarni P., Eisdorfer J.T. (2021). An Autonomous Molecular Bioluminescent Reporter (AMBER) for voltage imaging in freely moving animals. Adv. Biol..

[78] Oh J., Lee C., Kaang B.K. (2019). Imaging and analysis of genetically encoded calcium indicators linking neural circuits and behaviors. Korean J. Physiol. Pharmacol..

[79] Grynkiewicz G., Poenie M., Tsien R.Y. (1985). A new generation of Ca^2+^ indicators with greatly improved fluorescence properties. J. Biol. Chem..

[80] Barreto-Chang O.L., Dolmetsch R.E. (2009). Calcium imaging of cortical neurons using Fura-2 AM. J. Vis. Exp..

[81] Joseph P. (1994). Practical Aspects of Measuring [Ca^2+^] with Fluorescent Indicators. Methods Cell Biol..

[82] Minta A., Kao J.P., Tsien R.Y. (1989). Fluorescent indicators for cytosolic calcium based on rhodamine and fluorescein chromophores. J. Biol. Chem..

[83] Tada M., Takeuchi A., Hashizume M., Kitamura K., Kano M. (2014). A highly sensitive fluorescent indicator dye for calcium imaging of neural activity in vitro and in vivo. Eur. J. Neurosci..

[84] Broussard G.J., Liang R., Tian L. (2014). Monitoring activity in neural circuits with genetically encoded indicators. Front. Mol. Neurosci..

[85] Mank M., Griesbeck O. (2008). Genetically encoded calcium indicators. Chem. Rev..

[86] Miyawaki A., Llopis J., Heim R., McCaffery J.M., Adams J.A., Ikura M., Tsien R.Y. (1997). Fluorescent indicators for Ca^2+^ based on green fluorescent proteins and calmodulin. Nature.

[87] Horikawa K., Yamada Y., Matsuda T., Kobayashi K., Hashimoto M., Matsu-ura T., Miyawaki A., Michikawa T., Mikoshiba K., Nagai T. (2010). Spontaneous network activity visualized by ultrasensitive Ca^2+^ indicators, yellow Cameleon-Nano. Nat. Methods.

[88] Nakai J., Ohkura M., Imoto K. (2001). A high signal-to-noise Ca^2+^ probe composed of a single green fluorescent protein. Nat. Biotechnol..

[89] Tian L., Hires S.A., Mao T., Huber D., Chiappe M.E., Chalasani S.H., Petreanu L., Akerboom J., McKinney S.A., Schreiter E.R. (2009). Imaging neural activity in worms, flies and mice with improved GCaMP calcium indicators. Nat. Methods.

[90] Chen T.W., Wardill T.J., Sun Y., Pulver S.R., Renninger S.L., Baohan A., Schreiter E.R., Kerr R.A., Orger M.B., Jayaraman V. (2013). Ultrasensitive fluorescent proteins for imaging neuronal activity. Nature.

[91] Zhao Y., Araki S., Wu J., Teramoto T., Chang Y.F., Nakano M., Abdelfattah A.S., Fujiwara M., Ishihara T., Nagai T. (2011). An expanded palette of genetically encoded Ca^2+^ indicators. Science.

[92] Wu J., Prole D.L., Shen Y., Lin Z., Gnanasekaran A., Liu Y., Chen L., Zhou H., Chen S.W., Usachev Y.M. (2014). Red fluorescent genetically encoded Ca^2+^ indicators for use in mitochondria and endoplasmic reticulum. Biochem. J..

[93] Dana H., Mohar B., Sun Y., Narayan S., Gordus A., Hasseman J.P., Tsegaye G., Holt G.T., Hu A., Walpita D. (2016). Sensitive red protein calcium indicators for imaging neural activity. eLife.

[94] Baird G.S., Zacharias D.A., Tsien R.Y. (1999). Circular permutation and receptor insertion within green fluorescent proteins. Proc. Natl. Acad. Sci. USA.

[95] Nagai T., Sawano A., Park E.S., Miyawaki A. (2001). Circularly permuted green fluorescent proteins engineered to sense Ca^2+^. Proc. Natl. Acad. Sci. USA.

[96] Doronin D., Barykina N., Subach O., Sotskov V., Plusnin V., Ivleva O., Isaakova E., Varizhuk A., Pozmogova G., Malyshev A. (2018). Genetically encoded calcium indicator with NTnC-like design and enhanced fluorescence contrast and kinetics. BMC Biotechnol..

[97] Subach O.M., Sotskov V.P., Plusnin V.V., Gruzdeva A.M., Barykina N.V., Ivashkina O.I., Anokhin K.V., Nikolaeva A.Y., Korzhenevskiy D.A., Vlaskina A.V. (2020). Novel genetically encoded bright positive calcium indicator NCaMP7 based on the mNeonGreen fluorescent protein. Int. J. Mol. Sci..

[98] Lambert T.J., Waters J.C. (2014). Assessing camera performance for quantitative microscopy. Nat. Cell Biol..

[99] Zecevic D., Djurisic M., Cohen L.B., Antic S., Wachowiak M., Falk C.X., Zochowski M.R. (2003). Imaging nervous system activity with voltage-sensitive dyes. Curr. Protoc. Neurosci..

[100] Phil Brooks F., Davis H.C., Wong-Campos J.D., Cohen A.E. (2024). Optical constraints on two-photon voltage imaging. Neurophotonics.

[101] Wilt B.A., Fitzgerald J.E., Schnitzer M.J. (2013). Photon shot noise limits on optical detection of neuronal spikes and estimation of spike timing. Biophys. J..

[102] Sjulson L., Miesenbock G. (2007). Optical recording of action potentials and other discrete physiological events: A perspective from signal detection theory. Am. J. Physiol..

[103] Kiepas A., Voorand E., Mubaid F., Siegel P.M., Brown C.M. (2020). Optimizing live-cell fluorescence imaging conditions to minimize phototoxicity. J. Cell Sci..

[104] Bai L., Cong L., Shi Z., Zhao Y., Zhang Y., Lu B., Zhang J., Xiong Z.Q., Xu N., Mu Y. (2024). Volumetric voltage imaging of neuronal populations in the mouse brain by confocal light-field microscopy. Nat. Methods.

[105] Sheppard C.J., Gan X., Gu M., Roy M. (2006). Signal-to-noise ratio in confocal microscopes. Handbook of Biological Confocal Microscopy.

[106] Homma R., Baker B.J., Jin L., Garaschuk O., Konnerth A., Cohen L.B., Zecevic D. (2009). Wide-field and two-photon imaging of brain activity with voltage-and calcium-sensitive dyes. Philos. Trans. R. Soc. B.

[107] Wu J., Liang Y., Chen S., Hsu C.L., Chavarha M., Evans S.W., Shi D., Lin M.Z., Tsia K.K., Ji N. (2020). Kilohertz two-photon fluorescence microscopy imaging of neural activity in vivo. Nat. Methods.

[108] Li X., Li Y., Zhou Y., Wu J., Zhao Z., Fan J., Deng F., Wu Z., Xiao G., He J. (2023). Real-time denoising enables high-sensitivity fluorescence time-lapse imaging beyond the shot-noise limit. Nat. Biotechnol..

[109] Kaur S., Tang Z.F., McMillen D.R. (2025). A framework to enhance the signal-to-noise ratio for quantitative fluorescence microscopy. PLoS ONE.

[110] Tian T., Yuan Y., Mitra S., Gyongy I., Nolan M.F. (2022). Single photon kilohertz frame rate imaging of neural activity. Adv. Sci..

[111] Zhu M.H., Jang J., Milosevic M.M., Antic S.D. (2021). Population imaging discrepancies between a genetically-encoded calcium indicator (GECI) versus a genetically-encoded voltage indicator (GEVI). Sci. Rep..

[112] Pinkard H., Corbin K., Krummel M.F. (2016). Spatiotemporal rank filtering improves image quality compared to frame averaging in 2-photon laser scanning microscopy. PLoS ONE.

[113] Weigert M., Schmidt U., Boothe T., Müller A., Dibrov A., Jain A., Wilhelm B., Schmidt D., Broaddus C., Culley S. (2018). Content-aware image restoration: Pushing the limits of fluorescence microscopy. Nat. Methods.

[114] Mukamel E.A., Nimmerjahn A., Schnitzer M.J. (2009). Automated analysis of cellular signals from large-scale calcium imaging data. Neuron.

[115] Gabbay M., Brennan C., Kaplan E., Sirovich L. (2000). A principal components-based method for the detection of neuronal activity maps: Application to optical imaging. NeuroImage.

[116] Eom M., Han S., Park P., Kim G., Cho E.S., Sim J., Lee K.H., Kim S., Tian H., Böhm U.L. (2023). Statistically unbiased prediction enables accurate denoising of voltage imaging data. Nat. Methods.

[117] Kamran S.A., Moghnieh H., Hossain K.F., Bartlett A., Tavakkoli A., Drumm B.T., Sanders K.M., Baker S.A. (2024). Automated denoising software for calcium imaging signals using deep learning. Heliyon.

[118] Dalangin R., Jia B.Z., Qi Y., Aggarwal A., Sakoi K., Drobizhev M., Molina R.S., Patel R., Abdelfattah A.S., Zheng J. (2025). Far-red fluorescent genetically encoded calcium ion indicators. Nat. Commun..

[119] Liu P., Grenier V., Hong W., Muller V.R., Miller E.W. (2017). Fluorogenic targeting of voltage-sensitive dyes to neurons. J. Am. Chem. Soc..

[120] Gest A.M., Lazzari-Dean J.R., Ortiz G., Yaeger-Weiss S.K., Boggess S.C., Miller E.W. (2024). A red-emitting carborhodamine for monitoring and measuring membrane potential. Proc. Natl. Acad. Sci. USA.

[121] Fiala T., Wang J., Dunn M., Šebej P., Choi S.J., Nwadibia E.C., Fialova E., Martinez D.M., Cheetham C.E., Fogle K.J. (2020). Chemical targeting of voltage sensitive dyes to specific cells and molecules in the brain. J. Am. Chem. Soc..

[122] Bagur R., Hajnóczky G. (2017). Intracellular Ca^2+^ sensing: Its role in calcium homeostasis and signaling. Mol. Cell.

[123] DiGregorio D.A., Vergara J.L. (1997). Localized detection of action potential-induced presynaptic calcium transients at a Xenopus neuromuscular junction. J. Physiol..

[124] Milicevic K.D., Ivanova V.O., Brazil T.N., Varillas C.A., Zhu Y.M., Andjus P.R., Antic S.D. (2025). The Impact of Optical Undersampling on the Ca^2+^ Signal Resolution in Ca^2+^ Imaging of Spontaneous Neuronal Activity. J. Integr. Neurosci..

[125] Peng L., Zou P. (2023). Supertemporal resolution imaging of membrane potential via stroboscopic microscopy. Chem. Biomed. Imaging.

[126] Platisa J., Ye X., Ahrens A.M., Liu C., Chen I.A., Davison I.G., Tian L., Pieribone V.A., Chen J.L. (2023). High-speed low-light in vivo two-photon voltage imaging of large neuronal populations. Nat. Methods.

[127] Berthelon X., Chenegros G., Finateu T., Ieng S.H., Benosman R. (2018). Effects of cooling on the SNR and contrast detection of a low-light event-based camera. IEEE Trans. Biomed. Circuits Syst..

[128] Rasnik I., French T., Jacobson K., Berland K. (2013). Electronic cameras for low-light microscopy. Meth. Cell Biol..

[129] Lu S., Gao Y., Geng X., Guan Y. (2022). Peltier thermoelectric cooler improves both the signal-to-noise ratio and warm-up time of high-power LED induced fluorescence detector and application to aflatoxins. Anal. Chim. Acta.

[130] Carter B.D., Ashley M.C.B. (1991). A Peltier-Cooled CCD Camera. Publ. Astron. Soc. Aust..

[131] Holst G.C., Lomheim T.S. (2007). CMOS/CCD Sensors.

[132] Jonkman J., Brown C.M., Wright G.D., Anderson K.I., North A.J. (2020). Tutorial: Guidance for quantitative confocal microscopy. Nat. Protoc..

[133] Widenhorn R., Rest A., Blouke M.M., Berry R.L., Bodegom E. (2007). Computation of dark frames in digital imagers. Sensors, Cameras, and Systems for Scientific/Industrial Applications VIII, Proceedings of the IS&T/SPIE Electronic Imaging.

[134] Mubaid F., Kaufman D., Wee T.L., Nguyen-Huu D.S., Young D., Anghelopoulou M., Brown C.M. (2019). Fluorescence microscope light source stability. Histochem. Cell Biol..

[135] Xia C., Zhu D., Hu Y., Wang S., Guan J., Hou L., Shen Q., Jiang H. (2025). A multi-loop auto-locking system with fully digital electronics for ultra-stable laser. Rev. Sci. Instrum..

[136] Van Deun A., Chonde T., Gumusboga M., Rienthong S. (2008). Performance and acceptability of the FluoLED Easy™ module for tuberculosis fluorescence microscopy. Int. J. Tuberc. Lung Dis..

[137] Waters J.C., Wittmann T. (2014). Concepts in quantitative fluorescence microscopy. Methods in Cell Biology.

[138] Tompkins N., Fraden S. (2016). An inexpensive programmable illumination microscope with active feedback. Am. J. Phys..

[139] Baird T.R., Kaufman D., Brown C.M. (2014). Mercury free microscopy: An opportunity for core facility directors. J. Mol. Biol..

[140] Wiederschain G.Y. (2011). The Molecular Probes handbook. A guide to fluorescent probes and labeling technologies. Biochem.

[141] Kim B.B., Wu H., Hao Y.A., Pan M., Chavarha M., Zhao Y., Westberg M., St-Pierre F., Wu J.C., Lin M.Z. (2022). A red fluorescent protein with improved monomericity enables ratiometric voltage imaging with ASAP3. Sci. Rep..

[142] Wilson T. (2011). Resolution and optical sectioning in the confocal microscope. J. Microsc..

[143] Ching-Roa V.D., Huang C.Z., Giacomelli M.G. (2024). Suppression of Subpixel Jitter in Resonant Scanning Systems With Phase-locked Sampling. IEEE Trans. Med. Imaging.

[144] Akondi V., Kowalski B., Burns S.A., Dubra A. (2020). Dynamic distortion in resonant galvanometric optical scanners. Optica.

[145] Xu L., Tian X., Li X., Shang G., Yao J. (2011). Geometric distortion correction for sinusoidally scanned images. Meas. Sci. Technol..

[146] Xiong S., Peng L., Gu L., Zou P., Ji W. (2025). High pixel throughput voltage imaging based on repetitive optical selective exposure (ROSE). Optica.

[147] Colville M.J., Park S., Zipfel W.R., Paszek M.J. (2019). High-speed device synchronization in optical microscopy with an open-source hardware control platform. Sci. Rep..

[148] Salzberg B., Grinvald A., Cohen L., Davila H., Ross W. (1977). Optical recording of neuronal activity in an invertebrate central nervous system: Simultaneous monitoring of several neurons. J. Neurophysiol..

[149] London J., Zecevic D., Cohen L. (1987). Simultaneous optical recording of activity from many neurons during feeding in Navanax. J. Neurosci..

[150] Voigtländer B., Coenen P., Cherepanov V., Borgens P., Duden T., Tautz F.S. (2017). Low vibration laboratory with a single-stage vibration isolation for microscopy applications. Rev. Sci. Instrum..

[151] Wang L., Wang J., Liu S., Li A., Sun H., Liu X., Wang C. (2025). Design of a new type of high-speed scanning galvanometer structure and scanning control strategy. Mech. Syst. Signal Process..

[152] Yoo H.W., Ito S., Schitter G. (2016). High speed laser scanning microscopy by iterative learning control of a galvanometer scanner. Control Eng. Pract..

[153] Kowalski B., Akondi V., Dubra A. (2021). Correction of non-uniform angular velocity and sub-pixel jitter in optical scanning. Opt. Express.

[154] Pnevmatikakis E.A., Giovannucci A. (2017). NoRMCorre: An online algorithm for piecewise rigid motion correction of calcium imaging data. J. Neurosci. Methods.

[155] Lorenz K.S., Salama P., Dunn K.W., Delp E.J. (2012). Digital correction of motion artefacts in microscopy image sequences collected from living animals using rigid and nonrigid registration. J. Microsc..

[156] Hutton B.F., Braun M. (2003). Software for image registration: Algorithms, accuracy, efficacy. Semin. Nucl. Med..

[157] Hattori R., Komiyama T. (2022). PatchWarp: Corrections of non-uniform image distortions in two-photon calcium imaging data by patchwork affine transformations. Cell Rep. Methods.

[158] Rubart M. (2004). Two-photon microscopy of cells and tissue. Circ. Res..

[159] So P.T., Dong C.Y., Masters B.R., Berland K.M. (2000). Two-photon excitation fluorescence microscopy. Annu. Rev. Biomed. Eng..

[160] Prevedel R., Ferrer Ortas J., Kerr J.N., Waters J., Breckwoldt M.O., Deneen B., Monje M., Soyka S.J., Venkataramani V. (2025). Three-photon microscopy: An emerging technique for deep intravital brain imaging. Nat. Rev. Neurosci..

[161] Monakhov M.V., Matlashov M.E., Colavita M., Song C., Shcherbakova D.M., Antic S.D., Verkhusha V.V., Knöpfel T. (2020). Screening and cellular characterization of genetically encoded voltage indicators based on near-infrared fluorescent proteins. ACS Chem. Neurosci..

[162] Matlashov M.E., Shcherbakova D.M., Alvelid J., Baloban M., Pennacchietti F., Shemetov A.A., Testa I., Verkhusha V.V. (2020). A set of monomeric near-infrared fluorescent proteins for multicolor imaging across scales. Nat. Commun..

[163] Shemetov A.A., Monakhov M.V., Zhang Q., Canton-Josh J.E., Kumar M., Chen M., Matlashov M.E., Li X., Yang W., Nie L. (2021). A near-infrared genetically encoded calcium indicator for in vivo imaging. Nat. Biotechnol..

[164] Liu P., Miller E.W. (2019). Electrophysiology, unplugged: Imaging membrane potential with fluorescent indicators. Acc. Chem. Res..

[165] Song C., Matlashov M.E., Shcherbakova D.M., Antic S.D., Verkhusha V.V., Knöpfel T. (2024). Characterization of two near-infrared genetically encoded voltage indicators. Neurophotonics.

[166] Akemann W., Sasaki M., Mutoh H., Imamura T., Honkura N., Knöpfel T. (2013). Two-photon voltage imaging using a genetically encoded voltage indicator. Sci. Rep..

[167] Chamberland S., Yang H.H., Pan M.M., Evans S.W., Guan S., Chavarha M., Yang Y., Salesse C., Wu H., Wu J.C. (2017). Fast two-photon imaging of subcellular voltage dynamics in neuronal tissue with genetically encoded indicators. eLife.

[168] Grienberger C., Giovannucci A., Zeiger W., Portera-Cailliau C. (2022). Two-photon calcium imaging of neuronal activity. Nat. Rev. Methods Primers.

[169] Zong W., Obenhaus H.A., Skytøen E.R., Eneqvist H., de Jong N.L., Vale R., Jorge M.R., Moser M.B., Moser E.I. (2022). Large-scale two-photon calcium imaging in freely moving mice. Cell.

[170] Kuhn B., Roome C.J. (2019). Primer to voltage imaging with ANNINE dyes and two-photon microscopy. Front. Cell. Neurosci..

[171] Brooks F.P., Gong D., Davis H.C., Park P., Qi Y., Cohen A.E. (2025). Photophysics-informed two-photon voltage imaging using FRET-opsin voltage indicators. Sci. Adv..

[172] Andersson, Baechi, Hoechl, Richter (1998). Autofluorescence of living cells. J. Microsc..

[173] Reichman J. (2000). Handbook of Optical Filters for Fluorescence Microscopy.

[174] Jun Y.W., Kim H.R., Reo Y.J., Dai M., Ahn K.H. (2017). Addressing the autofluorescence issue in deep tissue imaging by two-photon microscopy: The significance of far-red emitting dyes. Chem. Sci..

[175] Zheng W., Wu Y., Li D., Qu J.Y. (2008). Autofluorescence of epithelial tissue: Single-photon versus two-photon excitation. J. Biomed. Opt..

[176] Sadashivaiah V., Tippani M., Page S.C., Kwon S.H., Bach S.V., Bharadwaj R.A., Hyde T.M., Kleinman J.E., Jaffe A.E., Maynard K.R. (2023). SUFI: An automated approach to spectral unmixing of fluorescent multiplex images captured in mouse and post-mortem human brain tissues. BMC Neurosci..

[177] Eigenfeld M., Kerpes R., Whitehead I., Becker T. (2022). Autofluorescence prediction model for fluorescence unmixing and age determination. Biotechnol. J..

[178] Jiang Y., Sha H., Liu S., Qin P., Zhang Y. (2023). AutoUnmix: An autoencoder-based spectral unmixing method for multi-color fluorescence microscopy imaging. Biomed. Opt. Express.

[179] Evans S.W., Shi D.Q., Chavarha M., Plitt M.H., Taxidis J., Madruga B., Fan J.L., Hwang F.J., van Keulen S.C., Suomivuori C.M. (2023). A positively tuned voltage indicator for extended electrical recordings in the brain. Nat. Methods.

[180] Abdelfattah A.S., Zheng J., Singh A., Huang Y.C., Reep D., Tsegaye G., Tsang A., Arthur B.J., Rehorova M., Olson C.V. (2023). Sensitivity optimization of a rhodopsin-based fluorescent voltage indicator. Neuron.

[181] St-Pierre F., Marshall J.D., Yang Y., Gong Y., Schnitzer M.J., Lin M.Z. (2014). High-fidelity optical reporting of neuronal electrical activity with an ultrafast fluorescent voltage sensor. Nat. Neurosci..

[182] Berezin M.Y., Achilefu S. (2010). Fluorescence lifetime measurements and biological imaging. Chem. Rev..

[183] Brinks D., Klein A.J., Cohen A.E. (2015). Two-photon lifetime imaging of voltage indicating proteins as a probe of absolute membrane voltage. Biophys. J..

[184] Park J., Gao L. (2024). Advancements in fluorescence lifetime imaging microscopy Instrumentation: Towards high speed and 3D. Curr. Opin. Solid State Mater. Sci..

[185] Knisley S.B., Justice R.K., Kong W., Johnson P.L. (2000). Ratiometry of transmembrane voltage-sensitive fluorescent dye emission in hearts. Am. J. Physiol. Heart Circ. Physiol..

[186] Lee P., Quintanilla J.G., Alfonso-Almazan J.M., Galán-Arriola C., Yan P., Sánchez-Gonzalez J., Perez-Castellano N., Pérez-Villacastín J., Ibanez B., Loew L.M. (2019). In vivo ratiometric optical mapping enables high-resolution cardiac electrophysiology in pig models. Cardiovasc. Res..

[187] Carandini M., Shimaoka D., Rossi L.F., Sato T.K., Benucci A., Knöpfel T. (2015). Imaging the awake visual cortex with a genetically encoded voltage indicator. J. Neurosci..

[188] Xie M.E., Adam Y., Fan L.Z., Böhm U.L., Kinsella I., Zhou D., Rozsa M., Singh A., Svoboda K., Paninski L. (2021). High-fidelity estimates of spikes and subthreshold waveforms from 1-photon voltage imaging in vivo. Cell Rep..

[189] Diaspro A., Chirico G., Usai C., Ramoino P., Dobrucki J. (2006). Photobleaching. Handbook of Biological Confocal Microscopy.

[190] Dynes J.L., Yeromin A.V., Cahalan M.D. (2023). Photoswitching alters fluorescence readout of jGCaMP8 Ca^2+^ indicators tethered to Orai1 channels. Proc. Natl. Acad. Sci. USA.

[191] Xu F., Shi D.Q., Lau P.M., Lin M.Z., Bi G.Q. (2018). Excitation wavelength optimization improves photostability of ASAP-family GEVIs. Mol. Brain.

[192] Bai J., Koos D.S., Stepanian K., Fouladian Z., Shayler D.W., Aparicio J.G., Fraser S.E., Moats R.A., Cobrinik D. (2023). Episodic live imaging of cone photoreceptor maturation in GNAT2-EGFP retinal organoids. Dis. Models Mech..

[193] Boudreau C., Wee T.L., Duh Y.R., Couto M.P., Ardakani K.H., Brown C.M. (2016). Excitation light dose engineering to reduce photo-bleaching and photo-toxicity. Sci. Rep..

[194] Chakrova N., Canton A.S., Danelon C., Stallinga S., Rieger B. (2016). Adaptive illumination reduces photobleaching in structured illumination microscopy. Biomed. Opt. Express.

[195] Wang Y., Ravasio C., Zhou Y., Han X. (2025). Prolonged Single Neuron Voltage Imaging in Behaving Mammals. bioRxiv.

[196] Vicente N.B., Zamboni J.E.D., Adur J.F., Paravani E.V., Casco V.H. (2007). Photobleaching correction in fluorescence microscopy images. J. Phys. Conf. Ser..

[197] Miura K. (2020). Bleach correction ImageJ plugin for compensating the photobleaching of time-lapse sequences. F1000Research.

[198] Hakonen A., Hulth S. (2008). A high-precision ratiometric fluorosensor for pH: Implementing time-dependent non-linear calibration protocols for drift compensation. Anal. Chim. Acta.

[199] Rühl P., Nair A.G., Gawande N., Dehiwalage S.N., Münster L., Schönherr R., Heinemann S.H. (2024). An Ultrasensitive Genetically Encoded Voltage Indicator Uncovers the Electrical Activity of Non-Excitable Cells. Adv. Sci..

[200] Sayresmith N.A., Saminathan A., Sailer J.K., Patberg S.M., Sandor K., Krishnan Y., Walter M.G. (2019). Photostable voltage-sensitive dyes based on simple, solvatofluorochromic, asymmetric thiazolothiazoles. J. Am. Chem. Soc..

[201] Yan P., Acker C.D., Zhou W.L., Lee P., Bollensdorff C., Negrean A., Lotti J., Sacconi L., Antic S.D., Kohl P. (2012). Palette of fluorinated voltage-sensitive hemicyanine dyes. Proc. Natl. Acad. Sci. USA.

[202] Romano S.A., Pérez-Schuster V., Jouary A., Boulanger-Weill J., Candeo A., Pietri T., Sumbre G. (2017). An integrated calcium imaging processing toolbox for the analysis of neuronal population dynamics. PLoS Comput. Biol..

[203] Briggs J.K., Jin E., Merrins M.J., Benninger R.K. (2025). CRISP: Correlation-refined image segmentation process. BMC Bioinform..

[204] Woolfe F., Gerdes M., Bello M., Tao X., Can A. (2010). Autofluorescence removal by non-negative matrix factorization. IEEE Trans. Image Process..

[205] Gao Z., Ling Z., Liu W., Han K., Zhang H., Hua X., Botchwey E.A., Jia S. (2025). Fluorescence microscopy through scattering media with robust matrix factorization. Cell Rep..

[206] Zhou P., Resendez S.L., Rodriguez-Romaguera J., Jimenez J.C., Neufeld S.Q., Giovannucci A., Friedrich J., Pnevmatikakis E.A., Stuber G.D., Hen R. (2018). Efficient and accurate extraction of in vivo calcium signals from microendoscopic video data. eLife.

[207] Fiala T., Sulzer D., Sames D. (2025). Seeing the Spikes: The Future of Targetable Synthetic Voltage Sensors. ACS Chem. Neurosci..

[208] Lee S., Kang B.E., Song Y.K., Baker B.J. (2022). A trafficking motif alters GEVI activity implicating persistent protein interactions at the membrane. Biophys. Reps..

[209] Baker C.A., Elyada Y.M., Parra A., Bolton M.M. (2016). Cellular resolution circuit mapping with temporal-focused excitation of soma-targeted channelrhodopsin. eLife.

[210] Piatkevich K.D., Bensussen S., Tseng H.a., Shroff S.N., Lopez-Huerta V.G., Park D., Jung E.E., Shemesh O.A., Straub C., Gritton H.J. (2019). Population imaging of neural activity in awake behaving mice. Nature.

[211] Grenier V., Walker A.S., Miller E.W. (2015). A small-molecule photoactivatable optical sensor of transmembrane potential. J. Am. Chem. Soc..

[212] Leong L.M., Shin S.C., Frankiv N., Rhee J.K., Kim H., Seong J., Woo J., Han K., Storace D.A., Baker B.J. (2025). Modulating Chromophore Flexibility in GEVIs through Threonine-Based Molecular Switches Reveals an Influence of Membrane Curvature on Protein Activity. ACS Sens..

[213] Sepehri Rad M., Cohen L.B., Braubach O., Baker B.J. (2018). Monitoring voltage fluctuations of intracellular membranes. Sci. Rep..

[214] Fan L.Z., Nehme R., Adam Y., Jung E.S., Wu H., Eggan K., Arnold D.B., Cohen A.E. (2018). All-optical synaptic electrophysiology probes mechanism of ketamine-induced disinhibition. Nat. Methods..

[215] Adam Y., Kim J.J., Lou S., Zhao Y., Xie M.E., Brinks D., Wu H., Mostajo-Radji M.A., Kheifets S., Parot V. (2019). Voltage imaging and optogenetics reveal behaviour-dependent changes in hippocampal dynamics. Nature.

[216] Onoue S., Kawamura K., Igarashi N., Zhou Y., Fujikawa M., Yamada H., Tsuda Y., Seto Y., Yamada S. (2008). Reactive oxygen species assay-based risk assessment of drug-induced phototoxicity: Classification criteria and application to drug candidates. J. Pharm. Biomed. Anal..

[217] Icha J., Weber M., Waters J.C., Norden C. (2017). Phototoxicity in live fluorescence microscopy, and how to avoid it. BioEssays.

[218] El-Khoury P.Z., Tarnovsky A.N., Schapiro I., Ryazantsev M.N., Olivucci M. (2009). Structure of the photochemical reaction path populated via promotion of CF2I2 into its first excited state. J. Phys. Chem. A.

[219] Preuss S., Stein W. (2013). Comparison of two voltage-sensitive dyes and their suitability for long-term imaging of neuronal activity. PLoS ONE.

[220] Wang D., Zhang Z., Chanda B., Jackson M.B. (2010). Improved probes for hybrid voltage sensor imaging. Biophys. J..

[221] Song C., Do Q.B., Antic S.D., Knöpfel T. (2017). Transgenic strategies for sparse but strong expression of genetically encoded voltage and calcium indicators. Int. J. Mol. Sci..

[222] Poburko D., Santo-Domingo J., Demaurex N. (2011). Dynamic regulation of the mitochondrial proton gradient during cytosolic calcium elevations. J. Biol. Chem..

[223] Yang Y., Liu N., He Y., Liu Y., Ge L., Zou L., Song S., Xiong W., Liu X. (2018). Improved calcium sensor GCaMP-X overcomes the calcium channel perturbations induced by the calmodulin in GCaMP. Nat. Commun..

[224] Kulkarni R.U., Vandenberghe M., Thunemann M., James F., Andreassen O.A., Djurovic S., Devor A., Miller E.W. (2018). In vivo two-photon voltage imaging with sulfonated rhodamine dyes. ACS Cent. Sci..

[225] Ahrens K.F., Heider B., Lee H., Isacoff E.Y., Siegel R.M. (2012). Two-photon scanning microscopy of in vivo sensory responses of cortical neurons genetically encoded with a fluorescent voltage sensor in rat. Front. Neural Circuits.

[226] Grenier V., Daws B.R., Liu P., Miller E.W. (2019). Spying on neuronal membrane potential with genetically targetable voltage indicators. J. Am. Chem. Soc..

[227] Akemann W., Lundby A., Mutoh H., Knöpfel T. (2009). Effect of voltage sensitive fluorescent proteins on neuronal excitability. Biophys. J..

[228] McMahon S.M., Jackson M.B. (2018). An inconvenient truth: Calcium sensors are calcium buffers. Trends Neurosci..

[229] Geng J., Tang Y., Yu Z., Gao Y., Li W., Lu Y., Wang B., Zhou H., Li P., Liu N. (2022). Chronic Ca^2+^ imaging of cortical neurons with long-term expression of GCaMP-X. eLife.

[230] Kang B.E., Baker B.J. (2016). Pado, a fluorescent protein with proton channel activity can optically monitor membrane potential, intracellular pH, and map gap junctions. Sci. Rep..

[231] Lee S., Geiller T., Jung A., Nakajima R., Song Y.K., Baker B.J. (2017). Improving a genetically encoded voltage indicator by modifying the cytoplasmic charge composition. Sci. Rep..

[232] Kang B.E., Lee S., Baker B.J. (2019). Optical consequences of a genetically-encoded voltage indicator with a pH sensitive fluorescent protein. Neurosci. Res..

